# A Metazoan/Plant-like Capping Enzyme and Cap Modified Nucleotides in the Unicellular Eukaryote *Trichomonas vaginalis*

**DOI:** 10.1371/journal.ppat.1000999

**Published:** 2010-07-15

**Authors:** Augusto Simoes-Barbosa, Robert P. Hirt, Patricia J. Johnson

**Affiliations:** 1 Department of Microbiology, Immunology, and Molecular Genetics, University of California, Los Angeles, Los Angeles, California, United States of America; 2 Institute for Cell and Molecular Biosciences, Newcastle University, Newcastle upon Tyne, United Kingdom; Yale School of Public Health, United States of America

## Abstract

The cap structure of eukaryotic messenger RNAs is initially elaborated through three enzymatic reactions: hydrolysis of the 5′-triphosphate, transfer of guanosine through a 5′-5′ triphosphate linkage and N7-methylation of the guanine cap. Three distinctive enzymes catalyze each reaction in various microbial eukaryotes, whereas the first two enzymes are fused into a single polypeptide in metazoans and plants. In addition to the guanosine cap, adjacent nucleotides are 2′-*O*-ribose methylated in metazoa and plants, but not in yeast. Analyses of various cap structures have suggested a linear phylogenetic trend of complexity. These findings have led to a model in which plants and metazoa evolved a two-component capping apparatus and modification of adjacent nucleotides while many microbial eukaryotes maintained the three-component system and did not develop modification of adjacent nucleotides. Here, we have characterized a bifunctional capping enzyme in the divergent microbial eukaryote *Trichomonas vaginalis* using biochemical and phylogenetic analyses. This unicellular parasite was found to harbor a metazoan/plant-like capping apparatus that is represented by a two-domain polypeptide containing a C-terminus guanylyltransferase and a cysteinyl phosphatase triphosphatase, distinct from its counterpart in other microbial eukaryotes. In addition, *T. vaginalis* mRNAs contain a cap 1 structure represented by m^7^GpppAmpUp or m^7^GpppCmpUp; a feature typical of metazoan and plant mRNAs but absent in yeast mRNAs. Phylogenetic and biochemical analyses of the origin of the *T. vaginalis* capping enzyme suggests a complex evolutionary model where differential gene loss and/or acquisition occurred in the development of the RNA capping apparatus and cap modified nucleotides during eukaryote diversification.

## Author Summary

The protozoan parasite *Trichomonas vaginalis* is the cause of the most common non-viral sexually transmitted disease worldwide. Evolutionary analyses place *Trichomonas* in a super group called the Excavata, which includes the kinetoplastids and is highly divergent from fungi, metazoa and plants. Despite the vast evolutionary distances that separate these different eukaryotic lineages, a simplified view of eukaryotic evolution based on the complexity of nucleotide modifications at the 5′ end of mRNAs and the distribution of different types of enzymatic apparatus that confer these modifications has been proposed. Our analyses of the *T. vaginalis* capping enzyme challenges this view and provides the first example of a two-component capping apparatus typically found in metazoa and plants in a protozoan. The 5′-end nucleotide structure of *T. vaginalis* mRNAs is also shown to contain additional modified nucleotides, similar to that observed for metazoan and plant mRNAs and unlike that found in most eukaryotic microbes and fungi. Evolutionary analyses of the *T. vaginalis* capping enzyme indicates that this multicellular type capping apparatus may have come into existence earlier than previously thought.

## Introduction

The 5′ cap is a unique feature of eukaryotic messenger RNAs (mRNA) and eukaryotic viruses not found on eubacterial and archaeal RNAs [1]. The addition of a m^7^G cap structure, or the cap 0 nucleotide, occurs co-transcriptionally via three consecutive reactions executed by the capping enzymatic apparatus: (i) hydrolysis of the 5′-triphosphate of nascent pre-mRNAs to a diphosphate by RNA 5′ triphosphatase (TPase), (ii) capping of the diphosphate end with GMP by the RNA guanylyltransferase (GTase) and (iii) methylation of the GpppN cap by RNA guanine-7- methyltransferase (MTase). The RNA cap is involved in multiple cellular functions including splicing, nucleocytoplasmic export, mRNA turnover and translation initiation [2], [3].

In addition to the N7-methylated guanosine cap, the 1st and 2nd adjacent nucleotides may be 2′O-ribose methylated after transcription [4] forming cap 1 and cap 2 structures, respectively. In contrast to the cap 0, the role of cap 1 and cap 2 modified nucleotides is unclear. Their presence may reveal a phylogenetic trend with increasing levels of complexity in multicellular eukaryotes [4], [5]. mRNAs in multiple species of yeast have been shown to contain only a cap 0 whereas the adjacent 5′ nucleotides of most multicellular eukaryotic mRNAs are further modified to form cap 1 and/or cap 2 structures [4], [6]. Thus far trypanosomes are the only protists to have their mRNA cap structures examined and these were found to contain a hypermodified cap 4 structure m^7^Gpppm_2_^6^AmpAmpCmpm^3^Um [7]. The unconventional method of providing mRNAs with cap modification via *trans*-splicing of a splice leader RNA at their 5′ ends confers this unique cap structure to trypanosome mRNAs [8], [9]. Recently, enzymes involved in cap 4 formation have been identified [10]–[12], although the role of these different cap nucleotide modifications remains to be elucidated [11], [13].

The N7-methylated guanosine cap (cap 0) is a structural consensus in all eukaryotic mRNAs unlike the different levels of modification of subsequent nucleotides observed in different organisms. The type and structural organization of the guanosine cap enzymatic apparatus that confers the cap 0 to the 5′end of mRNAs has been examined in a wide variety of eukaryotes [14]–[18], including the divergent protists *Giardia* [19] and trypanosomes [20]–[22]. An interesting evolutionary scenario has emerged [1], which contradicts [23] current models derived from multiple eukaryotic phylogenetic analyses [24]. This scenario dictates a multicellular (eg. metazoa and plants) versus a microbial eukaryotic pattern (e.g. fungi and microbial eukaryotes). The majority of investigated microbial eukaryotic species possess a three-component capping system (TPase, GTase and MTase) while metazoan and plants encode a two-component system with the fusion of TPase and GTase polypeptides and a separate MTase [1].

In contrast to structural conservation of GTases, the TPases appear to have evolved from different protein ancestors [1]. TPases found in multicellular eukaryotes contain the cysteinyl-phosphatase superfamily motif HCXXXXXR(S/T) named phosphate-binding loop or ‘p-loop’ (TPasePL). This enzyme catalyses a two-step phosphoryl transfer in which the conserved cysteine attacks the γ-phosphorus of the 5′-triphosphate on the nascent RNA to form a covalent protein-cysteinyl-S-phosphate intermediate producing a 5′-diphosphate RNA product [17], [25]. The enzyme-bound phosphate is then hydrolyzed and liberated as inorganic phosphate. In contrast, all investigated microbial eukaryotic TPases to date share structural organization with metal-dependent phosphohydrolases (TPaseMDP) and have different structural configurations and enzymatic characteristics [26]. A current model of the mRNA capping system evolution in eukaryotes suggests that a ‘transitional state organism’ would contain both types of TPases [1]. The fusion of the TPasePL-GTase was followed by a secondary loss or complete divergence of the TPaseMDP, and the last common ancestor (LCA) of plants and metazoans would carry the fused TPasePL-GTase version only. Although this model is supported by the distribution of these enzymes in several microbial eukaryotes [1], and is consistent with eukaryote phylogenies based on few genes supporting a metazoan-plant relationship [23], it is incongruent with phylogenomic and multiple single gene phylogenies [24], [27]. However this mRNA capping centric view of eukaryote phylogeny does not preclude the occurrence of differential gene loss/gain during eukaryotic evolution.

Here, we have characterized the guanosine cap enzymatic apparatus of *Trichomonas vaginalis*, a divergent microbial eukaryote, that is a member of the Parabasalia and the super-group Excavata [24], [28]. In contrast to microbial eukaryotes, including other members of the Excavata, the diplomonad *G. lamblia* [19] and kinetoplastids [20], [22] *T. vaginalis* has a dual TPasePL-GTase capping enzyme (TvCE) resembling the metazoan-plant type, as previously predicted from genome analyses [29]. Moreover, we have demonstrated that *T. vaginalis* mRNAs contain a complex cap structure with a canonical m^7^G and adjacent modified nucleotides. Phylogenetic analyses of the GTase domain only and TPasePL-GTase alignments are consistent with a common origin of the *T. vaginalis* and metazoan-plant enzymes, which suggest that the TPasePL-GTase system is likely to be more ancient then previously thought and that complex scenarios of independent gene loss and/or gain events across various eukaryotic lineages may have taken place.

## Results

**Figure 1:**
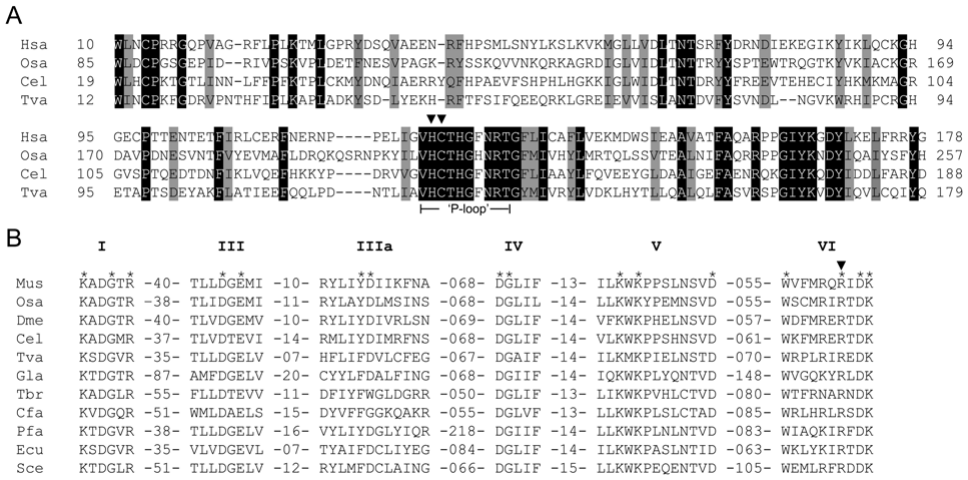
Sequence alignments of capping enzymes guanylyltransferase and triphosphatase. *Homo sapiens* (Hsa), *Saccharomyces cerevisiae* (Sce), *Mus musculus* (Mus), *Drosophila melanogaster* (Dme), *Oryza sativa* (Osa), *Caenorhabditis elegans* (Cel), *Plasmodium falciparum* (Pfa), *Encephalitozoon cuniculi* (Ecu), *Crithidia fasciculata* (Cfa), *Trypanosoma brucei gambiense* (Tbr), *Giardia lamblia* (Gla) and *T. vaginalis* (Tva) capping enzymes are shown. **A.** Sequence alignment of TvCE TPase reveals conservation with ‘metazoa/plant sequences and presence of the ‘P-loop’ active site. Identity and similarity are indicated in black and gray boxes, respectively. H125S and C126S substitutions in TvCE are indicated by arrowheads. **B.** GTase signature motifs conserved in all examined enzymes are indicated (I, III, IIIa, IV, V and VI). Numbers indicate the number of amino acid residues separating the motifs in different proteins. Essential amino acids for yeast (Sce) GTase are denoted by asterisks. The R526A substitution in TvCE is indicated by an arrowhead.

We have demonstrated previously that *T. vaginalis* mRNAs have a 5′-end protection that can be removed by pyrophosphatase treatment, and that these mRNAs partially precipitate with a monoclonal anti-trimethylguanosine antibody [30]. Therefore, similar to other eukaryotes this divergent protist must harbor an enzymatic capping apparatus. The *T. vaginalis* genome database (www.trichdb.org) [31] was screened using BLASTp analysis using available homologs for capping enzymes found in protists, yeast, plants and metazoans. As we previously reported based on analyses of the *T. vaginalis* genome [29] the same putative *T. vaginalis* capping enzyme gene (locus tag TVAG_187730, RefSeq accession XP_001327945.1, named here TvCE) is identified in searches conducted using the GTase enzyme found in many microbial eukaryotes and those using the fused TPasePL-GTase from plants and metazoans. No genes were identified in searches using TPaseMDP sequences. Both TPasePL-GTase functional domains in TvCE were conserved relative to the human capping enzyme in protein domain analyses as those from other metazoans, plants, green algae and a choanoflagellate, the latter being a member of the Choanomonada: microbial eukaryotes closely related to metazoan [24], [28] (Figure 1 & Figure S1). TvCE shares 30% identity and 47% similarity to the human capping enzyme and also has its TPasePL-domain fused to the N-terminus of a GTase domain (Figure S1). Strikingly, the TvCE cysteinyl-phosphatase superfamily motif HCXXXXXR(S/T) is 100% identical to the human sequence (Figure 1A). Furthermore, TvCE contained the six conserved peptide motifs (I, III, IIIa, IV, V and VI) at the C-terminus that comprise the active site for GTP binding and nucleotidyl transfer of GTase [32] (Figure 1B). Individual BLASTp searches with either the entire TvCE protein, the N-terminal domain encompassing the TPasePL domain (residues 1–258) or the two C-terminal domains (GTase, residues 259–441 and 442–561) identified by protein domain analyses, recovered as top hits animal sequences (see Data S1). These data indicate that *T. vaginalis* may have a two-component capping system similar to metazoan and plants.

**Figure 2:**
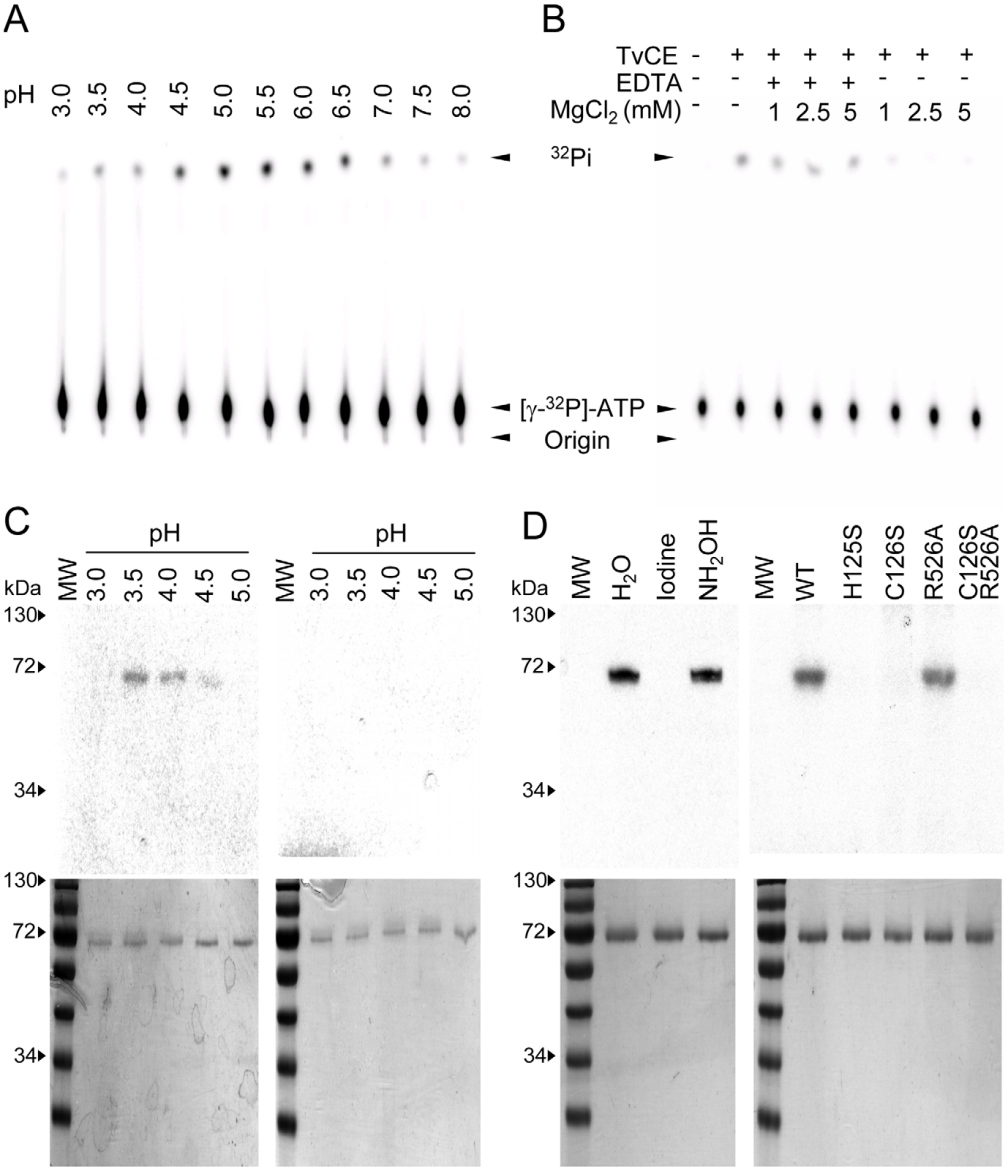
Triphosphatase activity of TvCE. **A.** Activity at various pHs. **B.** Effect of MgCl_2_ and EDTA on activity. Products were resolved on TLC plates and detected by autoradiography. Arrows denote the origin of loading, substrate ([γ-^32^P]ATP) and product (^32^Pi). **C.** Detection of the TvCE-cysteinyl-S-phosphate formation. Labeling with [γ-^32^P]ATP (left panel) and [α-^32^P]ATP (right panel) compared from pH 3.0–5.0. ^32^P-labeled TvCE detected by SDS-PAGE and autoradiography and TvCE protein loading controls flanked by pre-stained molecular weight markers (PageRuler prestained protein ladder, Fermentas) are shown in top and bottom panels, respectively. **D.** Effect of iodine and NH_2_OH on thiophosphate linkage formation (left panels) and TvCE-cysteinyl-S-phosphate formation of TvCE wild-type (WT) and mutant (H125S; C126S; R526A; C126S & R526A) proteins (right panels). ^32^P-labeled TvCE detected by SDS-PAGE and autoradiography (top panels) and TvCE protein loading controls (bottom panels) are shown. The purified protein is observed as a single band of the predicted ∼71 kDa molecular mass in both C and D.

**Figure 3:**
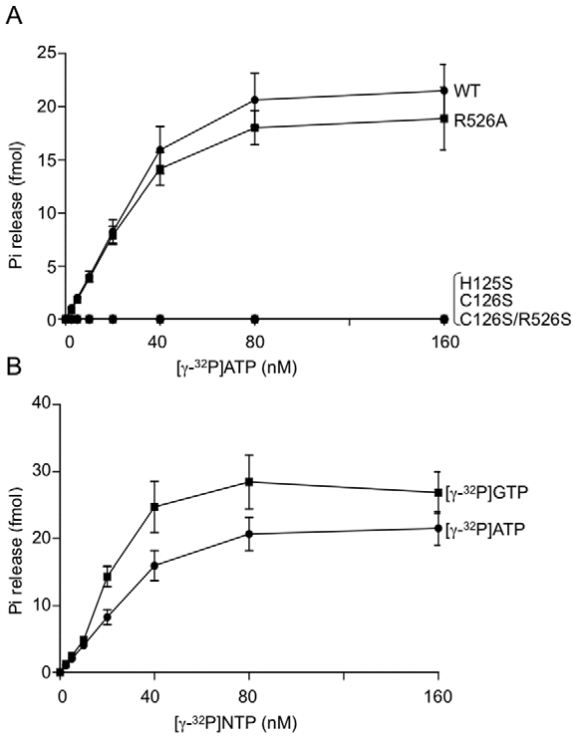
Triphosphatase activity of recombinant TvCE. **A.** Enzymatic activity of wild-type (WT) (filled circle) and mutants [R526A (filled square), H125S (filled triangle), C126S (open triangleD), C126S/R526S (open square)] was compared across a range of [γ-^32^P]ATP concentration. **B.** Enzymatic activity of wild-type TvCE was measured across a range of [γ-^32^P]ATP (filled circle) and [γ-^32^P]GTP (filled square) concentration for comparison of relative substrate affinity. Products were resolved on TLC plates and quantified by liquid scintillation counting.

To examine the enzymatic activity of recombinant TvCE we attempted to express either the full-length protein or each domain separately. We found that the single GTase domain was insoluble and that the TPase, although soluble, was inactive. Therefore, the full-length recombinant TvCE was used to analyse enzyme activity. The capping enzyme TPase specifically hydrolyses the γ-phosphate from the 5′-terminus of RNAs allowing its activity to be monitored by either the production of inorganic phosphate or ATP-ADP conversion. The TPase activity of recombinant TvCE was tested in the absence of metals at varying pH and the protein was found to be active in pH ranging from 4.5 to 6.5 (Figure 2A). Addition of 1–5 mM MgCl_2_ inhibited TvCE TPase activity and addition of EDTA could reverse this inhibition (Figure 2B). Activity in an acidic pH range and inhibition by MgCl_2_ are typical of a cysteinyl-phosphatase TPase and are not observed in classic microbial eukaryotic TPases [1]. In mammalian cysteinyl-phosphatase TPase, a transient phosphocysteine-enzyme intermediate can be trapped using a short incubation time at low temperature and acidic pH [17]. We asked whether TvCE also exhibits this property and found that the phospho-TvCE intermediate was detected in the presence of [γ^32^P]ATP, but not in the presence of [α^32^P]ATP as predicted, and within a restricted acidic pH (3.5–4.0) (Figure 2C). Phospholabeling of TvCE was lost after treatment with iodine but not hydroxylamine, supporting the presence of a thiophosphate linkage predicted to be at the C126 within the ‘P-loop’ (Figure 2D, left panel). To determine whether C126 is involved in this linkage, TvCE was subjected to specific amino acid mutations and tested for the ability to form a thiophosphate linkage. As shown in Figure 2D (right panel), serine substitution of either C126 or H125 abrogates formation of phospho-TvCE. When enzymatic affinity was compared across substrate concentration, we observed that P-loop mutants (H125S and C126S) and the double mutant (C126S and R526A) were inert to release γ-phosphate from ATP (Figure 3A), which is in agreement with the inability of these mutants to form a phosphocysteine-enzyme intermediate (Figure 2D). However, a mutation in the GTase domain alone (R526A) did not significantly affect TPase activity (Figure 3A). The utilization of ATP and GTP by TvCE as a function of nucleotide concentration is similar (Figure 3B). A relatively low conversion of the substrate is observed which may indicate that TvCE has either a low turnover rate or is partially inactivated during the 2-step purification. Together, these data indicate that C126 and H125 coordinate the cleavage and release of the γ-phosphate and demonstrate that TvCE has characteristics of a typical cysteinyl-phosphatase TPase [1].

**Figure 4:**
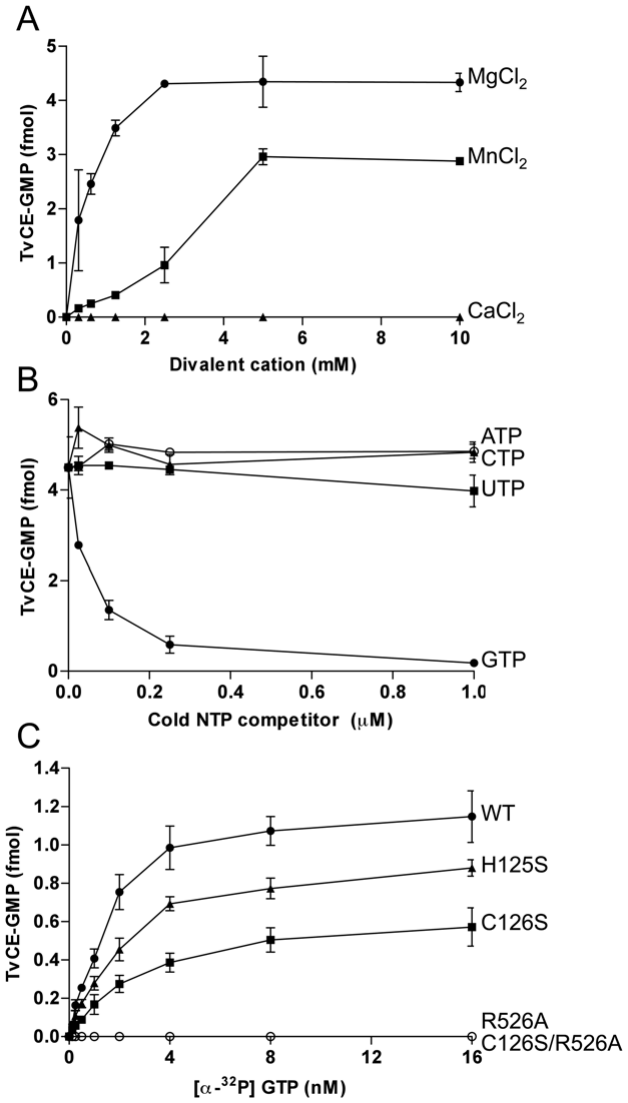
Guanylyltransferase activity recombinant TvCE. **A.** Effect of divalent cations Mg^2+^ (filled circle), Mn^2+^ (open square) and Ca^2+^ (filled triangle). **B.** Effect of competition with cold nucleotides ATP (open square), CTP (filled triangle), UTP (filled square) and GTP (filled circle). **C.** Enzymatic activity of wild-type (WT) (filled circle) and mutants [R526A (open square), H125S (filled triangle), C126S (filled square), C126S/R526S (open square)] was compared across a range of [α-^32^P]GTP concentration. Products were resolved on SDS-PAGE and quantified by liquid scintillation.

The capping enzyme GTase uses a ‘ping-pong’ reaction mechanism for nucleotidyl transfer through a covalent enzyme-(lysyl-*N*)-GMP intermediate [33]. This allows GTase activity to be detected by ^32^P transfer from [α-^32^P]GTP to the enzyme [14], [16], [18], [34]. To determine whether TvCE also uses this reaction mechanism, we incubated recombinant TvCE with [α-^32^P]GTP in the presence or absence of divalent cations. The formation of the SDS-stable ^32^P-labeled enzyme was then evaluated by SDS-PAGE (Figure 4). TvCE GTase activity was detected by [α-^32^P]GTP labeling in a broad pH window (data not shown) and required the presence of either MnCl_2_ or MgCl_2_. Calcium did not support activity (Figure 4A). Metal dependence, specifically Mn^2+^ and Mg^2+^, is a typical characteristic of an RNA capping GTase.

To determine the specificity of TvCE GTase, competition reactions were performed using increasing concentrations of cold NTPs. The recombinant TvCE GTase displayed an absolute specificity for a GTP substrate since ATP, CTP or UTP were not found to inhibit this enzyme (Figure 4B). Guanylation of TvCE is dependent on nucleotide concentration (Figure 4C). In accordance with previous reported structure/function studies [18], the single arginine substitution at GTase motif VI (R526A) abolished TvCE activity (Figure 4C). Interestingly, we also found that mutations in the TPase domain (H125S and C126S) significantly affected GTase activity, suggesting that these residues may exert a *cis*-structural effect on the GTase domain (Figure 4C). Using size exclusion chromatography, we found both TPase and GTase activities in a single discrete peak corresponding to ∼71 kDa indicating that TvCE is monomeric (Figure S2).

**Figure 5:**
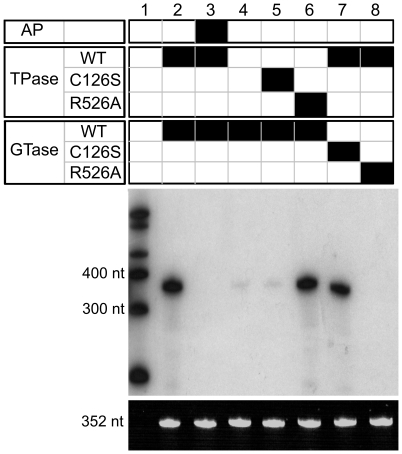
RNA capping activity of recombinant TvCE. Capping activity was detected in a two-step reaction, monitoring both TPase and GTase activities, using [α-^32^P]GTP and a 352 nt RNA substrate. Wild-type TvCE (WT), a TPase-inactive mutant (C126S) and a GTase-inactive mutant (R526S) were tested in the two-step reaction using various combinations as indicated in the black filled squares (top panel). A negative control reaction using the RNA substrate dephosphorylated by alkaline phosphatase prior to capping was included as indicated (AP). The final product was split equally and loaded on two polyacrylamide gels that were subsequently analyzed by autoradiography (middle panel) or ethidium bromide staining (bottom panel). Arrows denote molecular weights of radiolabeled RNA markers (Century Marker Plus, Ambion) and the RNA substrate.

In addition to using nucleotide substrates to assess the activity of TvCE we have also evaluated its ability to transfer GMP from GTP to the 5′ triphosphate end of an *in vitro* transcribed RNA, using the full-length enzyme with both active domains. As shown in Figure 5, TvCE is capable of labeling an RNA substrate provided this molecule harbors a 5′ triphosphate (lane 2). When the substrate is dephosphorylated by alkaline phosphatase digestion prior to incubation with TvCE, no transfer of [α-^32^P]GTP to the substrate is achieved (lane 3). In order for the GTase to transfer [α-^32^P]GMP from [α-^32^P]GTP to the 5′-end of the RNA substrate, the γ-phosphate must be first removed by the TPase (lane 4). When TPase or GTase activity is dependent on a TvCE mutant that is inactive in the corresponding domain, no labeling of the substrate is observed (lanes 5 & 8). On the other hand, when TPase or GTase activity is dependent on a TvCE mutant that inactivates the opposite domain, labeling of the substrate is achieved (lanes 6 & 7). These data are consistent with those characterizing individual domains using nucleotide substrates and TvCE mutants (Figure 3A and 4C) and demonstrate that capping of an RNA substrate strictly depends on the activity of both domains (Figure 5).

**Figure 6:**
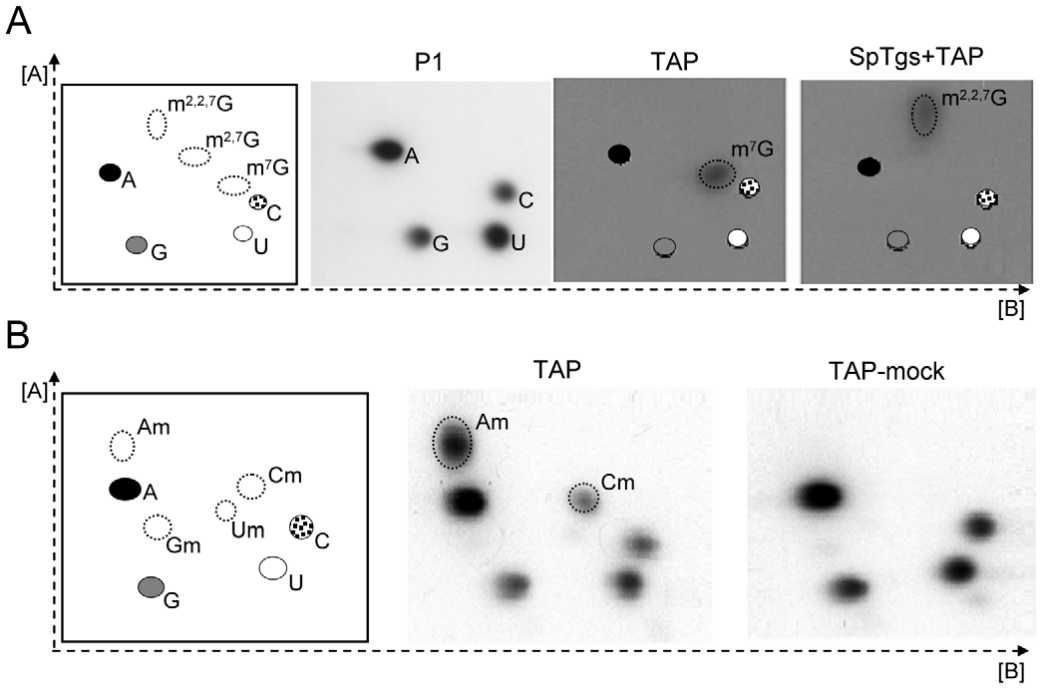
Detection of cap 0 and cap 1 modified nucleotides on *T. vaginalis* mRNA by 2D-TLC analysis. **A.** Detection of cap 0 modified nucleotide using *in vivo* ^32^P-labeled mRNA. The predicted migration of unmodified ribonucleotides (A, C, G and U) are shown in all panels for reference and left panel also denote modified ribonucleotide standards migration (dotted ovals). **P1** = nuclease P1 digestion; **TAP** = TAP treatment; **SpTgs+TAP** = SpTgs incubation prior to TAP treatment. **B.** Detection of cap 1-modified nucleotide. Unlabeled *T. vaginalis* mRNA either TAP treated (**TAP**) or mock-treated (**TAP-mock**) subjected to 5′-end labeling, nuclease P1 digestion and resolution on 2D-TLC.

The observed similarities between metazoa and *T. vaginalis* RNA capping enzymatic activities prompted us to investigate the mRNA cap structure in this organism. The nucleotides adjacent to the m^7^G-cap structure are methylated to different extents in eukaryotes. Metazoans can have 2′-*O*-ribose methylations of the first and second transcribed nucleotides forming the cap structure m^7^GpppNmpNmpNp, where the first transcribed nucleotide is an adenosine. However, yeast is found to have no modifications beyond the 5′ m^7^G cap [4], [6]. We developed a multi-step protocol to purify mRNA suitable for structural analysis of nucleotides (see Materials and Methods). None of the steps alone, including consecutive passages over poly-dT chromatography columns, was sufficient to remove abundant RNA species such as tRNA and rRNA (Figure S3). As these contaminants contain hypermodified nucleotides in relatively high abundance that interfered with our analysis, we found it necessary to also immunoprecipitate RNAs with anti-TMG (which cross-reacts with the 7-methyl guanosine cap of mRNAs) to remove uncapped RNA contaminants (Figure S3). As a result, *in vivo* ^32^P-labeled mRNA obtained by this purification protocol was shown to be free of contaminating rRNA and tRNA by gel electrophoresis (data not shown) and structural analysis of nucleotides after P1 digestion (Figure 6A and Figure S3). The abundance of adenosine and uridine, relative to guanosine and cytosine, in this heterogeneous mRNA population (Figure 6A) is consistent with the reported ∼65% AT content of *T. vaginalis* genes [29]. Digestion of the purified mRNA sample with Tobacco Acid Pyrophosphatase (TAP), which specifically hydrolyzes the phosphoric acid anhydride bonds in the triphosphate bridge of a 5′-end cap structure, released m^7^GMP (Figure 6A). The identity of this modified nucleotide was confirmed by demonstrating that it can be converted to m^2,2,7^GMP using *Schizosaccharomyces pombe* trimethylguanosine synthase or SpTgs (Figure 6A) [35], [36].

Next, a sample of unlabeled mRNA was used for analysis of the first transcribed nucleotide (position +1). RNAs were subjected to TAP or a TAP-mock treatment, alkaline phosphatase digestion and then 5′-end labeled using T4 polynucleotide kinase. These were then digested completely by nuclease P1, and nucleotides were resolved on 2D-TLC plates. Appearance of distinct spots presence only in the TAP treated sample should reveal whether the first transcribed mRNA nucleotide (position +1), which is protected by an m^7^G cap, is modified (Figure 6B). Our result demonstrated that *T. vaginalis* mRNAs have a typical metazoan 2′-*O*-ribose methylated cap 1 nucleotide. The cap 1 nucleotide is either an adenosine (80%) or a cytosine (20%) based on a comparison of the Am/Cm ratio (Figure 6B). This is in agreement with ∼75% of *T. vaginalis* protein-coding genes being preceded by the conserved initiator element (Inr) TCAT/_C_T/_A_ that dictates transcription initiation at the underlined adenosine [29], [37]. The four unmodified nucleotides (A, C, G and U) observed in both samples likely result from partially degraded RNAs that would not require TAP treatment to be 5′-end labeled or the presence of intact RNA contaminants that lack a cap.

**Figure 7:**
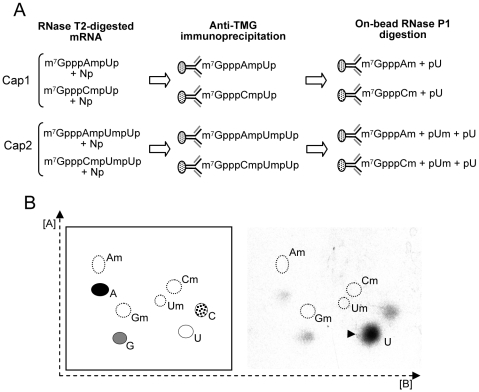
Evaluation of a cap 2 modified nucleotide in *T. vaginalis* mRNA by 2D-TLC analysis. **A.** Scheme of experimental approach. For simplicity, uridine is shown at position +2 and +3 as this is the most common nucleotide at these positions [28], [29]. The presence of cap 1 modified nucleotides Am or Cm (Figure 4B) predict the release of 3′-phosphate mononucleotides (Np), and 2 of 4 possible cap structures depending on the absence or presence of a cap 2 modified uridine upon RNase T2 digestion. The presence of only a cap 1 structure (top) predicts release of m^7^GpppAmpUp or m^7^GpppCmpUp; if a cap 2 is present (bottom) m^7^GpppAmpUmpUp or m^7^GpppCmpUmpUp is predicted. Subsequent anti-TMG immunoprecipitation and elution by RNase P1 digestion predicts release of only unmodified ribonucleotides or both unmodified and 2-*O*-ribose modified ribonucleotides in the absence or presence of a cap 2 ribonucleotide, respectively. **B.** Detection of unmodified ribonucleotides at +2 of *T. vaginalis* mRNAs using the scheme shown in **A.** and 2D-TLC (right). Migration standards are shown in left panel.

There is a strong bias for a uridine at positions +2 and +3 in most *T. vaginalis* mRNAs [37], [38]. To demonstrate whether the uridine +2 is 2′-*O*-ribose methylated forming a cap 2 structure, *in vivo* labeled mRNA was digested with RNase T2 prior to anti-TMG precipitation, the last step of the mRNA purification protocol (Figure 7A). 2′-*O*-ribose methylation of this nucleotide would render the adjacent 3′-5′ phosphodiester linkage resistant to RNase T2 as this enzyme cleaves RNA via 2′-3′phosphate cyclization. These samples were then immunoprecipitated with anti-TMG agarose beads and 3′-5′ phosphodiester linkages were cleaved by on-bead RNase P1 treatment, to restrict analysis to the cap 2 modified nucleotide, if present, and its adjacent cap 1 nucleotide. Released nucleotides were then analyzed by 2D-TLC. If nucleotide +2 is not 2′-*O*-ribose methylated only the cap 1 unmodified nucleotide, mostly uridine, would be released, compared to the release of both 2′-*O*-ribose methylated nucleotides (position +2) and the unmodified nucleotide (position +3) if a cap 2 modified nucleotide is present (Figure 7A). As a result, no modified nucleotides were detected indicating the absence of a cap 2 structure in *T. vaginalis* mRNAs (Figure 7B). As predicted the most abundant unmodified nucleotide detected was uridine. Although these data cannot exclude the presence of a cap 2 structure in a small subset of mRNAs, if present these are highly underrepresented.

**Figure 8:**
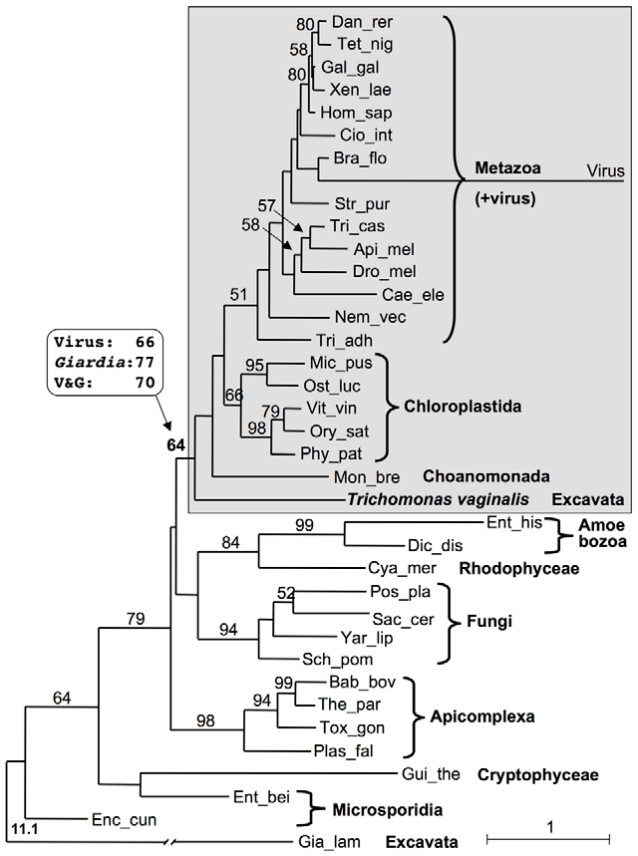
Phylogenetic analysis of TvCE. Protein maximum likelihood phylogeny recovered for 37 GTases functioning with TPaseMDP and TPasePL-GTases configured enzymes that maximizes taxonomic diversity. A clan of all TPasePL-GTases, that includes TvCE, is boxed and shaded. Numbers indicate the bootstrap proportion (BP) support values (>50% are shown). Boxed values list BP for alternative analyses where the indicated sequences were removed; V&G indicates removal of both the viral and *Giardia* sequences. The tree was rooted on *Giardia lamblia*, the most divergent sequence (broken branch to fit and true branch length value indicated). Removal of either one or the two most divergent sequences (*Giardia* and the iridovirus) did not affect the overall tree topology (see Data S1; Figure S4). Higher taxonomic terms [24], [28] are indicated. Species names abbreviated with the first three letters of the genus and species are all listed in full in Table S1 - with corresponding accession numbers. The scale bar indicates inferred number of substitutions per site.

**Figure 9:**
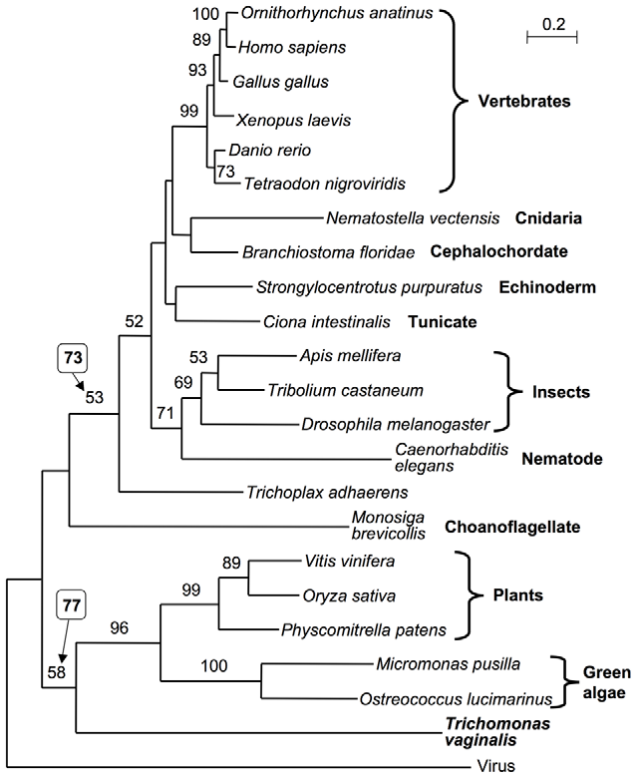
Phylogenetic analyses to maximize taxonomic diversity. Protein maximum likelihood phylogeny recovered for 23 TPasePL-GTases selected to maximize taxonomic diversity. The tree is rooted on the viral sequence and numbers indicate BP values >50%. Boxed values indicate BP for corresponding analyses conducted without the iridovirus sequence (see Data S1, Figure S4). The scale bar indicates inferred number of substitutions per site.

The unexpected finding of a metazoan/plant-like capping apparatus and a cap 1 modified nucleotide on *T. vaginalis* mRNAs led us to investigate the phylogeny of TvCE. A global taxa sampling of animal, fungal, plant, microbial and iridovirus GTases as well as a subset dataset including exclusively the TPasePL-GTase structural organization were aligned and subjected to protein maximum likelihood phylogenetic analyses (Figure 8 & Figure 9). Although generally poorly resolved, the global GTase phylogeny recovered the *T. vaginalis* sequence with modest support value (64% bootstrap proportion, BP but increasing to 70% and 77% when one or two of the most divergent sequences were removed) in a clan of exclusively TPasePL-GTase configured sequences, consistent with a common origin of their structural organization (Figure 8). The TPasePL-GTase maximum likelihood tree revealed that TvCE does not cluster with proteins from animals (Figure 9) and hence, at face value, does not support lateral gene transfer (LGT) between *T. vaginalis* or a Parabasalid ancestor and their animal hosts. These data are consistent with an ‘Unikonts’/‘Bikonts’ split [39] with on one hand plants and green algae forming a clan with *T. vaginalis* supported with 58% BP, raising to 77% in the absence of the iridovirus sequence (Figure 9), and the metazoan and choanoflagellates forming the other clan in line with phylogenomic data [24]. As the position of the eukaryotic root is currently unknown [39] it is not clear how the iridovirus sequence relates to the eukaryotic sequences; its divergent sequence makes inference of its position in both analyses tentative. Consistent with potential long-branch attraction (LBA) issues for this sequence, its removal in the TPasePL-GTase alignment improved the BP of several nodes including the one for the *T. vaginalis*-plant/green algae clan and the metazoan clan (Figure 9). Recoding the 20 amino acids into four categories, to allow optimization of the rate matrix and reduce composition heterogeneity between sequences and mitigate potential LBA artefacts, recovered a similar tree with reduced BP for the split between the TvCE-plant/algae clan and the metazoan clan in maximum likelihood analyses. Together our data favor a tree topology consistent with phylogenomics data [24] and cluster TvCE with plant and green algae sequences; however, LGT from a metazoan donor to a parabasalid cannot be strictly rejected due to lack of strong signal.

## Discussion

This is the first report of the functional characterization of a microbial eukaryote harboring a typical metazoan/plant-like capping apparatus containing a fused dual-functional TPasePL-GTase (TvCE). In addition to this unusual capping apparatus in *T. vaginalis*, mRNAs from this unicellular eukaryote were found to contain a cap 1 structure, another feature of metazoan mRNAs. The GTase domain of TvCE selectively binds GMP. The transfer of GMP to the 5′ triphosphate end of the RNA is strictly dependent on the prior removal of the γ-phosphate by the TPase activity of TvCE. The TPase domain of TvCE has typical features of a metazoan cysteinyl-phosphatase enzyme; it is active in the absence of metals as a bifunctional monomeric enzyme, prefers an acidic pH, and forms a phospho-enzyme through a cysteine-thiophosphate linkage within a conserved ‘P-loop’ active site which is necessary for phosphatase activity. An apparent *cis*-structural effect in TvCE was observed between the two active domains as a single amino acid change in the GTase domain results in a detectable reduction of TPase activity. We also observed that purification of the individual TvCE TPase domain resulted in an inactive enzyme. Together, these data suggest that the GTase domain can affect the activity of its neighboring TPase domain.

Analyses of the cap structure of mRNAs in this divergent microbial eukaryote revealed the presence of a canonical m^7^G cap 0 nucleotide, consistent with the presence of two conserved RNA (guanine-7) MTase genes in the *T. vaginalis* genome (http://trichdb.org) that could convert the guanosine at the 5′ end of the mRNA by TvCE to an m^7^G. We previously showed that *T. vaginalis* has an atypical trimethylguanosine synthase (TgS) that produces m^2,7^G from m^7^G RNA substrates [36]. These observations led us to speculate that *T. vaginalis* mRNAs might contain m^2,7^G caps. Contrary to this prediction, the analyses presented here demonstrate the presence of a canonical m^7^G cap nucleotide on *T. vaginalis* mRNAs.

A general comparison of cap structures among different eukaryotes suggested that complexity increases following a phylogenetic trend across the evolution of eukaryotes [5]. This is illustrated by the presence of a complex cap 2 structure on mRNAs of metazoans but only cap 0 on budding yeast mRNAs. Prior to the studies reported here, the unusual cap 4 in trypanosomatids [7]–[11] appeared to be the exception to this rule. *T. vaginalis* mRNAs which contain a conserved cap 1 structure composed primarily of m^7^GpppAmpUp or m^7^GpppCmpUp are now also exceptions. No cap 2 structure was detected in *T. vaginalis* mRNA and a strong bias to uridines at position +2 was observed, making this cap structure less complex than metazoans. It should also be noted however, that the use of a heterogeneous population of mRNA may have obscured the detection of a cap 2 nucleotide in a smaller subpopulation of mRNAs. Similarly, although we consider it unlikely, we cannot strictly preclude that immunoprecipitation of mRNAs using the anti-TMG antibody led to the exclusion of a subset of mRNAs with a cap 2 nucleotide or an alternative cap structure that are not efficiently bound by this antibody.

The observation that ∼80% of cap 1 nucleotides in *T. vaginalis* mRNAs are adenosines (A) while ∼20% are cytosines (C) indicates that transcription initiation by RNA polymerase II in this organism can occur at cytosine, as well as adenosine. In turn this suggests the presence of either unknown variants of the initiator (Inr) motif that surrounds the start site of transcription of *T. vaginalis* mRNAs [37], [38] or unrelated motifs that can direct transcription to initiate at a cytosine. This is consistent with our previous prediction that only ∼75% of *T. vaginalis* genes appear to use a classic Inr leading to transcription initiation at an A [29].

The structural organization of the mRNA caps enzyme machinery in eukaryotes has been considered a marker for inference of eukaryote phylogeny [1]. Fungi and most sampled microbial eukaryotes have separate TPaseMDP and GPase capping enzymes with the TPaseMDP structurally and mechanistically distinct from the TPasePL that is fused to a GTase in metazoan and plants. Thus, the acquisition of the metazoan-type dual-function enzyme after the divergence of unicellular and multicellular eukaryotes has been proposed [1]. However recent genome samplings have broadened the taxonomic diversity of TPasePL-GTase encoding taxa, which now includes green algae, a choanoflagellate and *T. vaginalis*. These additional data and our structural, functional and phylogenetic analyses of TvCE complicates the earlier simple dichotomy observed between metazoan-plants and microbial eukaryotes. Indeed, the proponent of this dichotomy acknowledged that “The scheme is certainly oversimplified…” due to poor taxa sampling [1].

Differential gene losses could explain the unusual presence of a metazoan/plant-like capping apparatus in *T. vaginalis*, green algae and choanoflagellates. The LCA of microbial eukaryotes, plants and animals may have contained the fused TPasePL-GTase and independent gene losses subsequently occurred in most currently sampled microbial eukaryotes, leaving *T. vaginalis*, green algae and choanoflagellates as rare microbial eukaryotes carrying this prototype. Notably the green algae and choanoflagellate TPasePL-GTase sequences were recovered in the expected clans as defined by phylogenomics [24], pushing the acquisition of this configuration among a likely microbial ancestor deeper in eukaryotic evolution. Alternatively, the LCA of plants/green algae and animals/choanoflagellates had the split system seen today in many microbial eukaryotes and subsequently lost this and acquired a new fused version. Similar but independent events would than be invoked for the acquisition of TvCE, leading to an enzyme with little sequence relatedness to its counterpart in plants and animals. However the global GTase phylogeny suggests that the GTase from the TPasePL-GTase fusion was shared by the LCA between *T. vaginalis*, plants/algae and choanoflagellate/animals; as expected if the gene was present in the LCA of all eukaryotes. Alternatively, the GTase for the taxa with TPasePL-GTase configurations was independently acquired by different lineages from similar sources with the same structural organization and/or *T. vaginalis* acquired TvCE by LGT. Existing phylogenies do not provide positive evidence for these scenarios. As Parabasalia and Diplomonads appear to be closely related within the excavates [24] an independent origin scenario of the TPasePL-GTase fusion could have been suggested if the TvCE GTase formed a clan with the *G. lamblia* GTase, however this was not found in our maximum likelihood phylogenetic analyses (Figure 8, Figure S4). These data clearly reinforce the importance of further genome sampling among various microbial eukaryotes and viruses before evolutionary hypotheses based on the mRNA capping system can be appropriately assessed and contrasted to the current hypothesis of eukaryote phylogeny [39]. Phylogenomic data obtained so far consistently provide evidence for at least six major eukaryotic lineages [24], [27] that do not match those suggested by the distribution of the mRNA capping enzymes [1]. Our phylogenetic analyses do not support LGT as the source of TvCE in *T. vaginalis* reducing the conflict between the distribution of the mRNA capping machinery and the six major lineages recovered by phylogenomics [39]. In contrast, relationships among microbial eukaryote GTases functioning with TPaseMDP seem more complex and in conflict with phylogenomic data in terms of major group relationships. For instance non-monophyly is observed for microsporidial GTases, which do not cluster with the Fungi, and the red algae cluster with the Amoebozoa. Could these be explained by LGT events as described for the acquisition of a trifunctional capping enzyme in a mimivirus, thought to be derived from its amoeba host [40]? Likewise the iridovirus TPasePL-GTase may have been acquired from an animal host. The evolution of capping enzymes in eukaryotes appears to have proceeded via multiple events that led to the independent loss and/or gain of genes in different lineages. Polarization of such events will require denser sampling of mRNA capping genes and additional robust independent phylogenetic analyses.

## Materials and Methods

### Expression and purification of recombinant TvCE

The ORF encoding TvCE (TVAG_187730) was cloned into *Escherichia coli* expression vector pET 200D- (Invitrogen), which adds a Histidine-tag at the N-terminus. TvCE was cloned either as a full length protein or as two separate domains. The full-length protein has a predicted molecular weight of 69 kDa (not including the histidine tag) and a pI of 6.9. A MUSCLE alignment of the protein predicted the TPase domain to reside between amino acids 1 and 254 and the GTase domain between amino acids 255 and 561. pET-200D constructs containing either the full-length or the TPase or GTase domain were transfected into *E. coli* strain BL21 as provided by the manufacturer (Invitrogen). 250 ml of bacteria cultures were grown to OD_600_ 0.4–0.5 and 3% (v/v) ethanol and 0.2 mM IPTG was added to induce protein expression. Incubation was continued for 18–20h at 18°C, shaking at 180 rpm. Induced cells were centrifuged and resuspended in 50 mM NaPO4 pH 6.0, 1 mg/ml of lysozyme and 1× Halt protease inhibitor cocktail (ThermoScientific). Lysis was achieved by sonication on ice and cell debris was removed by spinning samples at 16,000× g for 30 min. The presence of soluble expressed recombinant protein was evaluated by SDS-PAGE before loading it on a pre-equilibrated 5 ml Mono-S column (GE Healthcare). The column was washed with 10 volumes loading buffer minus lysozyme. Proteins were eluted with a 5 ml step gradient of NaCl (100, 200, 300, 400 and 500 mM) in the same buffer. Eluted fractions shown to contain rTvCE by SDS-PAGE analysis were pooled together and imidazole was added to 40 mM. The proteins were further fractionated using Ni chromatography (HisTrap; GE Healthcare), as recommended by the manufacturer. After the two-step purification, the final purified rTvCE (50–150 ug of protein) was dialyzed against 50 mM Tris pH 7.4, 100 mM NaCl, 2 mM DTT and 10% glycerol. PCR mutagenesis was carried out as described [41] and proteins were isolated using the 2-step purification scheme described above.

### TPase activity assays

To determine optimal pH of TvCE, 20 µl reactions containing 25 nM of full-length recombinant TvCE (flr-TvCE), 5 mM DTT, 160 nM [γ-^32^P]ATP were adjusted to 50 mM Tris-acetate (pH 7.0 and below) or 50 mM Tris-HCl (pH 7.5 and above). To evaluate metal dependence of TvCE TPase, 20 µl reactions containing 50 mM Tris-acetate pH 5.5, 350 nM of flr-TvCE, 5 mM DTT and 15 nM [γ-^32^P]ATP were performed in the presence of 0, 1, 2.5 and 5 mM MgCl_2_. These reactions were assays in either the presence of 0 or 20 mM EDTA for comparison. TvCE TPase nucleotide dependence was measured in 20 µl reactions containing 50 mM Tris-acetate pH 5.5, 5 mM DTT, a range of 2.5–160 nM [γ-^32^P]ATP or [γ-^32^P]GTP and 25 nM of flr-TvCE. For detection of phosphatase activity of TvCE, reactions were incubated at 37°C for 30 min, and ^32^Pi was detected on TLC plates after autoradiography as described [17]. Products were sliced from the TLC plastic plates for quantification by liquid scintillation. A mock reaction (minus enzyme) was done in parallel to account for spontaneous radiolysis of the substrate. TvCE TPase was tested for the formation of a covalent protein-cysteinyl-S-phosphate intermediate [17], [25]. To test the hypothesis that such an intermediate can be formed under acidic pH, a 10 µl reaction containing 50 mM Tris-acetate pH 3.0 to 5.0, 5 mM DTT, 160 nM [γ-^32^P]ATP and 25 nM of flr-TvCE was performed at 25°C for 15 seconds. One µl of this reaction was loaded on SDS-PAGE for Coomassie Blue staining and autoradiography. This analysis was carried out for flr-TvCE mutants, except that molar concentration of [γ-^32^P]ATP was increased to 330 nM, unincorporated nucleotides were removed by G-50 microcolumns (Amersham) and all sample contents were analyzed by SDS-PAGE. To confirm that TvCE is phosphor-labeled through formation of a thiophosphate linkage, chemical stability analysis was performed as previously described [17]. For this analysis, we compared thiophosphate linkage stability when phosphor-TvCE was treated with H_2_O, 100 mM NH_2_OH or 10 mM iodine.

### GTase activity assays

To evaluate metal specificity of TvCE GTPase, 10 µl reactions containing 50 mM Tris-acetate pH 7.0, 5 mM DTT, 20 nM [α-^32^P]GTP and 40 nM of full-length recombinant TvCE (flr-TvCE) were performed, varying the concentrations of either MgCl_2_, MnCl_2_ or CaCl_2_ from 0–10 mM. To evaluate substrate specificity of TvCE GTPase, a cold competition experiment was designed. 10 µl reactions containing 50 mM Tris-acetate pH 7.0, 5 mM DTT, 2.5 mM MgCl_2_, 100 nM [α-^32^P]GTP, 40 nM of recombinant TvCE were performed in the presence of 0–1 µM cold nucleotide competitor ATP, CTP, GTP or UTP. TvCE GTase dependence on nucleotide concentration was measured in 20 µl reactions containing 50 mM Tris-acetate pH 7.0, 5 mM DTT, a range of 0–16 nM [α-^32^P]GTP and 3 nM flr-TvCE. All reactions were incubated at 37°C for 30 min. and GTase activity was detected by the formation of the covalent enzyme-GMP intermediate [18]. The reaction product was detected on SDS-PAGE and autoradiography. The phosphor-labeled enzyme was sliced from gels and quantified by liquid scintillation.

### Measuring TvCE activity using a RNA substrate

A fragment containing 352 bp of *T. vaginalis* ß-tubulin was *in vitro* transcribed by T7 RNA polymerase and quantified as previously described [36], and used as a substrate for the full length recombinant TvCE in a two-step reaction. As a negative control, one RNA sample was dephosphorylated by alkaline phosphatase treatment (Apex, Epicentre) prior to TvCE RNA capping activity and purified by phenol/chloroform extraction and ethanol precipitation. The first step of the reaction, the removal of the γ-phosphate, was tested by incubating 250 ng of the RNA in a 50 µl reaction containing 50 mM Tris-acetate pH 5.5, 5 mM DTT, and 200 nM recombinant TvCE at 37°C for 30 min. The RNA was then purified by phenol/chloroform extraction and ethanol precipitation. Next, the RNA was resuspended in a 50 µl reaction containing 50 mM Tris-acetate pH 7.0, 5 mM DTT, 2.5 mM MgCl_2_, 90 nM [α-^32^P]GTP and 200 nM recombinant TvCE and incubated at 37°C for 30 min. The RNA was then purified as described above, split into equal part and analyzed in 7% polyacrylamide gels under denaturing conditions (Tris-Borate EDTA buffer and 8M urea). One sample was stained by ethidium bromide and the other was dried and exposed to X-ray film.

### Cap structure analysis

*T. vaginalis* strain T1, grown in TYM complete media [42] was subjected to *in vivo* labeling of RNAs. To achieve 12–18% total ^32^P incorporation, 1–5×10^8^ parasites were starved in the absence of phosphate-, serum-free DMEM and 1 mCi of phosphorus-32 radionuclide for 8–9 hours at 37°C. Cultures were mixed by inversion every hour during incubation. RNA was extracted and size-fractionated from ^32^P-labeled and unlabeled *T. vaginalis* cultures using the *mir*Vana PARIS Kit (Ambion). To determine the nucleotide structure of the cap of *T. vaginalis* mRNAs, a protocol to obtain a population of mRNAs free of the hypermodified ribosomal and transfer RNAs was developed. The high relative abundance of hypermodified nucleotides present in these RNA species interfered with detection of modified nucleotides found specifically on mRNAs in the presence of minor contamination by rRNA or tRNA. First the large-size RNA fraction (>200 nt) was isolated from cells to minimize contamination with small rRNA and tRNAs. The RNA is then passed by two consecutive rounds of poly dT purification (Promega). The eluted RNA was then concentrated by ethanol precipitation, followed by Terminator Exonuclease (Epicentre) digestion to degrade RNAs that contain a 5′-monophosphate (ex. rRNAs). The large-size RNAs were again isolated, and purified from partially degraded rRNAs and nucleotides. As a final step, the mRNAs were purified by immunoprecipitation using the mouse monoclonal anti-TMG (anti-trimethylguanosine agarose conjugate, CalBiochem) as described [30], taking advantage of the cross-reactivity of this antibody with the 7-methyl guanosine cap of mRNAs. The purity of the mRNA preparation protocol was assessed by electrophoresis and two-dimension thin layer chromatography (2D-TLC) analysis of *in vivo* labeled mRNAs after autoradiography. 2D-TLC was carried out using both combinations of organic solvents A, B and C, as previously described [36], [43]. To evaluate the presence of a 5′-end guanosine cap linked by a triphosphate bridge to the RNA, *in vivo* labeled mRNAs (∼10^5^ cpm) were digested with TAP (Epicentre) and analyzed by 2D-TLC. To confirm the identity of a possible m^7^G cap structure in this highly purified fraction of *T. vaginalis* mRNA, the sample was treated with *S. pombe* Tgs prior to TAP treatment. This enzyme converts m^7^G to m^2,2,7^G which can resolved by 2D-TLC [36]. To identify a possible nucleotide modification at position +1, mRNA was 5′-end labeled. The mRNA was digested or mock-digested with TAP, dephosphorylated by Alkaline Phosphatase (APex, Epicentre) and heat-inactivated, and labeled by PNK with [γ-^32^P]ATP. Between enzymatic treatments, RNA was purified by phenol/chloroform extraction and ethanol precipitation. TAP-digested or mock-digested samples were comparatively analyzed by 2D-TLC. Spots were quantified by liquid scintillation. To identify a possible nucleotide modification at position +2, we utilized *in vivo* labeled mRNAs. The protocol necessary to obtain mRNA free of detectable hypermodified nucleotides from ribosomal and transfer RNA, as described above, was followed except that before anti-TMG immunoprecipitation the RNA sample was concentrated by ethanol precipitation and digested with RNase T2 in a 20 µl reaction volume. Six µl of this reaction was then anti-TMG immunoprecipitated in a 0.6 ml end volume. After washes, the RNase T2-digested m^7^G capped mRNAs bound to the anti-TMG agarose beads were mildly digested with nuclease P1 in a 25 µl reaction volume for 2 h at 37°C under agitation. The anti-TMG beads were then recentrifuged, the supernatant was recovered and P1-digestion continued at 50°C for 16 h to completion. Five µl of this reaction were loaded on 2D-TLC plates for analysis. For 2D-TLC comparative maps, radiolabeled m^7^G, m^2,7^G and m^2,2,7^G standards were produced as previously reported [36]. 2′-*O*-ribose methylated nucleotide standards were produced by P1-digestion of a AmCmGmUm oligomer (Sigma-Proligo), and all were compared to previous reported maps [36], [43].

### General bioinformatics and phylogenetics

Sequences were extracted from protein databases following BLASTp searches at NCBI using default settings except for the sequence of the red algae *Cyanidioschyzon merolae* GTase which was obtained from the KEGG database. Selected sequences aligned for comparison and phylogenetic inferences are described in Data S1. Proteins structural organizations were investigated with SMART searching both SM and Pfam profiles [44]. For phylogenetics reference protein sequences were chosen from BLAST hit lists to maximize taxa diversity and sequences aligned with ClustalW [45]. SEAVIEW 4.0 [46] was used to visually check the alignment features and sites used for phylogenetic analyses were selected with the mask option. Sites with more then five indels were deleted as where divergent sites. The best-fitting models for the protein alignments were identified with Prottest 2.2 [47], which was invariably the LG rate matrix [48] with a gamma rate model “G” for across site rate variation. PhyML [49] was used within SEAVIEW to perform maximum likelihood phylogenetic inferences. The alpha shape parameter of the gamma rate model (four categories) was estimated using the BioNJ distance tree used as the starting tree in conjunction of both NNI and TPR branch swapping moves for further optimization. The optimal alpha shape parameter for site rate variation was then fixed and used for bootstrap analyses (100 replicates). In order to mitigate potential issues of composition bias and long branch attractions, the protein alignment were recoded for the TPasePL-GTase protein alignment, as described [24] with the 20 amino acids reduced to four categories implied by the JTT rate matrix ([A,N,G,T,P,S], [R,D,E,Q,K], [E,L,M,F,V] and [HWYC]). Following removal of invariant sites in the recoded alignment (410 sites were reduced to 316 sites), PhyML maximum likelihood analyses (GTR rate matrix with gamma model, both estimated) were performed and recovered similar trees as those based on the protein alignments. All alignments are available upon request.

## Supporting Information

Data S1 Bioinformatic data.
(0.03 MB DOC)

Figure S1 Structural organization of TvCE. **A.** SMART (http://smart.embl-heidelberg.de/) analyses identified the same three domains in TvCE found in other TPasePL-GTase proteins. The corresponding structural organization for the human sequence is shown for comparison. In the human sequence the pink polygon corresponds to a segment of low compositional complexity and vertical bars corresponds to intron positions. Values below bars indicate intron phase and values above the bars indicate the corresponding amino acid positions. Overlapping domains identified by SMART are not indicated. **B.** Position and e-values for the three identified domains (TPase: PTP_DSPc: GTase: mRNA cap enzyme; C: cap C domain) in the parasite and humans sequences with the most significant scores are tabulated for comparison.
(0.35 MB TIF)

Figure S2 Determination of the molecular weight of the active form of TvCE. The recombinant TvCE was subjected to size exclusion chromatography in a pre-calibrated 90–10 cm Superdex™ 200 column (GE). Molecular weight (MW) markers (Sigma): Soybean trypsin inhibitor (20 kDa), chicken egg albumin (∼44 kDa), bovine serum albumin (66 kDa), alcohol dehydrogenase (∼150 kDa), apoferritin (443 kDa) and thyroglobulin (∼669 kDa). For MW curve standardization, excluded volume (Vo) was estimated by dextran blue, and elution volume (Ve) for each protein was collected and calculated. A 2 ml mix containing 0.5 mg of each protein, 50mM Tris pH 6.0; 200 mM NaCl, 5mM DTT; 10% glycerol was loaded into the column. Ninety five fractions (2 ml each) were collected at 0.2 ml/min. **A.** MW curve and TvCE activity. The linear regression of the MW curve (dotted line) and its equation are shown along with GTase (filled circle) and TPase (open circle) activity curves. Each MW marker is indicated by size (filled square). The activity peak and the first and last fractions tested for TvCE activity are indicated by a circled number which corresponds to the fraction number (fractions 30–50). The y axis indicates the molecular weight values (left) and GTase and TPase activities (right), and the x axis indicates Ve/Vo. **B.** Western blot analysis of TvCE after size fractionation. Five microliters of fractions 2 to 90, as indicated, were analyzed by western blot with HisProbeTM-HRP (Pierce). The peak fraction 42, and every other fraction from 30 (∼440 kDa) to 50 (∼50 kDa), denoted by circles, were examined for TvCE activity as described in Methods.
(4.26 MB TIF)

Figure S3 2D-TLC resolution of P1-digested mRNA purified from *T. vaginalis*. Left panel, *in vivo*-labeled mRNA was prepared as described in Methods except that three rounds of oligo(dT) chromatography was performed but the final step of anti-TMG precipitation was omitted. Right panel, mRNA was prepared exactly as described in Methods. The final mRNA sample from each approach was then precipitated with ethanol and digested with nuclease P1 to completion. A total of 10,000 cpm of the digested RNA was resolved by 2D-TLC, as decribed in Methods, and resolved with solvents A and B (43) as indicated. Cold unmodified nucleotides (A, C, G and U) were loaded in each plate as denoted. As observed, even with one additional round of purification by oligo(dT) chromatography but without anti-TMG immunoprecipitation (left panel), the complete digestion of this mRNA sample produces the four unmodified nucleotides plus additional six detectable spots which seem to correspond to hypermodified nucleotides found in tRNAs as compared to 2D-TLC maps previously reported (43). However, when a final step of anti-TMG immunoprecipitation was included for the purification of mRNAs (right panel), the complete digestion of this sample resulted in the apperance of the four unmodified ribonucleotides only, the typical composition of mRNA molecules in a cell. Additionaly, the relative percentage of A/T (∼70%) is in accordance with A/T content of genes in this organism (29).
(1.17 MB TIF)

Figure S4 Alternative protein phylogenetic analyses of TvCE. **A.** Global phylogeny of GTases with the same alignment used to generate phylogeny depicted in Figure 8 with the iridovirus sequence removed. The arrow indicates the branch leading to all TPasePL-GTase configured sequences. **B.** Global phylogeny of GTases with the same alignmnent used to generate phylogeny depicted in Figure 8 with the *Giardia* sequence removed. The arrow indicates the branch leading to all TPasePL-GTase configures sequences. **C.** Same as in A with the *Giardia* sequence further removed. **D.** Phylogeny of TPasePL-GTase configured sequences with the same alignment used to generate the phylogeny depicted in Figure 9 with the iridovirus removed. In all trees the species names are abbreviated with the first three letters of the genus and species name (full names are listed in supplementary Table S1) and the LG model with G was used. Shown values are bootstrap proportions (%, 100 replicates), values >50% are shown. The alpha shape parameter was optimized first and fixed for the bootstrap analyses with NNI and TBR branch swapping for further optimizations. Scale bars represent the inferred number of changes per site.
(0.20 MB PDF)

Table S1 Full names of species used in phylogenetic analyses and accession numbers for all gene sequences used in the work described here.
(0.05 MB DOC)
