# Supplementary material for: A Metazoan/Plant-like Capping Enzyme and Cap Modified Nucleotides in the Unicellular Eukaryote Trichomonas vaginalis
Source: PLoS Pathog. 2010 Jul 15;6(7):e1000999. doi: 10.1371/journal.ppat.1000999 (PMC2904801; doi:10.1371/journal.ppat.1000999)
Supplement: Data S1 — Bioinformatic data. (0.03 MB DOC) [file ppat.1000999.s001.doc]

**Supporting Information (SI)**

**Summary of BlastP searches results with TvCE domains.**

Individual BlastP searches were performed with either the N-terminal segment encompassing the TPasePL domain (residues 1-258), the two C-terminal domains (GTase, residues 259-441 and 442-561) identified by SMART analyses (see Fig. 1), or the entire TvCE sequence. Selected information for the top hit for each respective BlastP search is listed below and includes the taxa name, the various scores generated for each BlastP alignment and the BlastP alignment.

**Database Name:** refseq_protein

**Program:** BLASTP 2.2.21+

**Date:** August 2009

>TvCE residues 1-258

MTDNKFTPPKRWINCPKFGDRVPNTHFIPLKAPLADKYSDLYEKHRFTFSIFQEEQRKLGREIEVVISLANTDVFYSVNDLNGVKWRHIPCRGHETAPTSDEYAKFLATIEEFQQLPDNTLIAVHCTHGFNRTGYMIVRYLVDKLHYTLLQALQLFASVRSPGIYKVDYIQVLCQIYQVDETQTLSELFHVPPSEIKNIKKPKWEFPDPSTLPHFRVEKPIVNETQTLENVGTHFRDSAITSEISKICSVNYGRFPGS

**Top hit:**

Rattus norvegicus (brown rat, ...) [rodents] taxid 10116

ref|NP_001101393.1| RNA guanylyltransferase and 5'-phospha... 134 6e-30

>ref|NP_001101393.1| Gene info RNA guanylyltransferase and 5'-phosphatase [Rattus norvegicus]

Length=597

Score = 134 bits (338), Expect = 6e-30, Method: Compositional matrix adjust.

Identities = 70/180 (38%), Positives = 105/180 (58%), Gaps = 6/180 (3%)

Query 1 MTDNKFTPPKRWINCPKFGDRVPNTHFIPLKAPLADKY-SDLYEKHRFTFSIFQEEQRKL 59

M NK P RW+NCP+ G V F+PLK L +Y S + E++RF S+ + L

Sbjct 1 MAYNKIPP--RWLNCPRRGQPVAG-RFLPLKTMLGPRYDSQVAEENRFHPSMLSNYLKSL 57

Query 60 GREIEVVISLANTDVFYSVNDLN--GVKWRHIPCRGHETAPTSDEYAKFLATIEEFQQLP 117

++ +++ L NT FY ND+ G+K+ + C+GH PT++ F+ E F +

Sbjct 58 KVKMSLLVDLTNTSRFYDRNDIEKEGIKYIKLQCKGHGECPTTENTETFIRLCERFNERS 117

Query 118 DNTLIAVHCTHGFNRTGYMIVRYLVDKLHYTLLQALQLFASVRSPGIYKVDYIQVLCQIY 177

LI VHCTHGFNRTG++I +LV+K+ +++ A+ FA R PGIYK DY++ L + Y

Sbjct 118 PPELIGVHCTHGFNRTGFLICAFLVEKMDWSIEAAVATFAQARPPGIYKGDYLKELFRRY 177

>TvCE residues 259-441

NPISITSENKMQLTQKRYLATYKSDGVRYFLYAFHKNTYLIDRKNSIRKVNVTLVTRKGQPMENTLLDGELVVSKGDDKPHFLIFDVLCFEGLNLTNHTWDLRMDYSKKGVVPFRKMYFNRNPGAFTREDFHIEEKLQWELQNIRDLEEYIMTKVTHDTDGAIFTPLDLEFVPGRCDQILKMK

**Top hit:**

Trichoplax adhaerens [placozoans] taxid 10228

ref|XP_002114529.1| hypothetical protein TRIADDRAFT_58446 ... 119 1e-25

>ref|XP_002114529.1| Gene info hypothetical protein TRIADDRAFT_58446 [Trichoplax adhaerens]

Length=615

Score = 119 bits (298), Expect = 1e-25, Method: Compositional matrix adjust.

Identities = 74/193 (38%), Positives = 107/193 (55%), Gaps = 12/193 (6%)

Query 260 PISITSENKMQLTQKRYLATYKSDGVRYFLYAFHKN--TYLIDRKNSIRKVNVTLVTRKG 317

P+S+T N L +RY ++K+DGVRY + HKN Y+IDR NSI K+ R

Sbjct 267 PVSLTYNNITLLRDRRYRVSWKADGVRYMML-IHKNDEIYMIDRNNSIFKIPHLKFPRGS 325

Query 318 ---QPMENTLLDGELVVSK-----GDDK-PHFLIFDVLCFEGLNLTNHTWDLRMDYSKKG 368

+ENTLLDGE+V+ K GD P +LI+D++CFE N+ N RM +K

Sbjct 326 DLNSHIENTLLDGEMVIDKVSTPNGDQYYPRYLIYDIICFEDENVGNKKQSERMAIIEKE 385

Query 369 VVPFRKMYFNRNPGAFTREDFHIEEKLQWELQNIRDLEEYIMTKVTHDTDGAIFTPLDLE 428

++ R R T+E F + K ++ ++ R + + KV H+TDG IF+P D

Sbjct 386 IISPRNQAAARGIVDKTKETFSVRNKQFFDAKDARYVLDTFTKKVFHETDGLIFSPEDEP 445

Query 429 FVPGRCDQILKMK 441

++PGRCD +LK K

Sbjct 446 YIPGRCDTVLKWK 458

>TvCE_442-561

PIELNSTDFKIQLHNGIYYMSVTNYVKNDEKFQENIPISILDFADGLGVKDGAICEAVLDLKKEDIENDPINCWFKAGWRPLRIREDKDTPNVYTTFAGVFKSIEDNINFDTIAKMFPKK

**Top hit:**

Trichoplax adhaerens [placozoans] taxid 10228

ref|XP_002114529.1| hypothetical protein TRIADDRAFT_58446 ... 45 0.001

>ref|XP_002114529.1| Gene info hypothetical protein TRIADDRAFT_58446 [Trichoplax adhaerens]

Length=615

Score = 45.8 bits (107), Expect = 0.001, Method: Compositional matrix adjust.

Identities = 40/127 (31%), Positives = 52/127 (40%), Gaps = 26/127 (20%)

Query 442 PIELNSTDFKIQL-----HNGIYYMSVTNYVK--NDEKFQENIPISILDFADGLGVKDGA 494

P ELN+ DFK+ L H + +V N ++ IP GL D

Sbjct 459 PAELNTVDFKLHLVKVEKHGCLPTKEARLHVGYGNSQRHVATIP------GKGLNKFDSK 512

Query 495 ICEAVLDLKKEDIENDPINCWFKAGWRPLRIREDKDTPNVYTTFAGVFKSIEDNINFDTI 554

I E LD K W+ LRIREDK PN ++TF V SI I DT+

Sbjct 513 IVECCLDGKTRT-------------WKILRIREDKAFPNAHSTFIAVCNSILMPITKDTL 559

Query 555 AKMFPKK 561

+ K+

Sbjct 560 YYVVEKR 566

>TvCE residues 1-561

**Top hit:**

Trichoplax adhaerens [placozoans] taxid 10228

ref|XP_002114529.1| hypothetical protein TRIADDRAFT_58446 ... 275 6e-72

>ref|XP_002114529.1| Gene info hypothetical protein TRIADDRAFT_58446 [Trichoplax adhaerens]

Length=615

GENE ID: 6755742 TRIADDRAFT_58446 | hypothetical protein [Trichoplax adhaerens]

(10 or fewer PubMed links)

Score = 275 bits (704), Expect = 6e-72, Method: Compositional matrix adjust.

Identities = 197/596 (33%), Positives = 301/596 (50%), Gaps = 68/596 (11%)

Query 2 TDNKFTPP--KRWINCPKFGDRVPNTHFIPLKAPLADKYSDLYE-KHRFTFSIFQEEQRK 58

T +KF+ P RW+ CP+ G+ V F+P K PL +Y++ + RF ++ +

Sbjct 3 TSDKFSLPIPPRWLKCPRRGNVVAGK-FLPFKVPLDSRYNEQIPIEDRFDINMLFQFTNA 61

Query 59 LGREIEVVISLANTDVFYSVNDL--NGVKWRHIPCRGHETAPTSDEYAKFLATIEEFQQL 116

+ ++I L NTD FYS +++ N + + +GH P+ D+ F+ +F Q

Sbjct 62 YKINLGLIIDLTNTDRFYSKSEVESNNAGYLKLRLKGHGEVPSPDQCTLFIEICMKFIQN 121

Query 117 PDNTLIAVHCTHGFNRTGYMIVRYLVDKLHYTLLQALQLFASVRSPGIYKVDYIQVLCQI 176

N++I +HCTHGFNRTG++I YL++K +++ AL+ FAS RSPGIYK Y++ L Q

Sbjct 122 NPNSVIGIHCTHGFNRTGFLICCYLIEKEDWSVQAALREFASARSPGIYKGYYMKELAQR 181

Query 177 YQVDETQTLSELFHVPPSEIKNIKKPKWEFPDP-------STLPHFRVEKPIVNETQTLE 229

Y + + ++ E+ P+W DP S+ + + ++ + + +E

Sbjct 182 YDPN-----GDFDYISAPEL-----PEWCNEDPHSDEEIVSSRKKRKRNEHLILDPKFME 231

Query 230 NVG--THFRDSAITSEISKICSVNYG----RFPGSNPISITSENKMQLTQKRYLATYKSD 283

V RD ++ S++ ++C G FPGS P+S+T N L +RY ++K+D

Sbjct 232 GVRGPVPVRDQSL-SDLQELCQEKCGWMEGGFPGSQPVSLTYNNITLLRDRRYRVSWKAD 290

Query 284 GVRYFLYAFHKN--TYLIDRKNSIRKVNVTLVTRKG---QPMENTLLDGELVVSK----- 333

GVRY + HKN Y+IDR NSI K+ R +ENTLLDGE+V+ K

Sbjct 291 GVRYMML-IHKNDEIYMIDRNNSIFKIPHLKFPRGSDLNSHIENTLLDGEMVIDKVSTPN 349

Query 334 GDDK-PHFLIFDVLCFEGLNLTNHTWDLRMDYSKKGVVPFRKMYFNRNPGAFTREDFHIE 392

GD P +LI+D++CFE N+ N RM +K ++ R R T+E F +

Sbjct 350 GDQYYPRYLIYDIICFEDENVGNKKQSERMAIIEKEIISPRNQAAARGIVDKTKETFSVR 409

Query 393 EKLQWELQNIRDLEEYIMTKVTHDTDGAIFTPLDLEFVPGRCDQILKMKPIELNSTDFKI 452

K ++ ++ R + + KV H+TDG IF+P D ++PGRCD +LK KP ELN+ DFK+

Sbjct 410 NKQFFDAKDARYVLDTFTKKVFHETDGLIFSPEDEPYIPGRCDTVLKWKPAELNTVDFKL 469

Query 453 QL-----HNGIYYMSVTNYV--KNDEKFQENIPISILDFADGLGVKDGAICEAVLDLKKE 505

L H + +V N ++ IP GL D I E LD K

Sbjct 470 HLVKVEKHGCLPTKEARLHVGYGNSQRHVATIP------GKGLNKFDSKIVECCLDGKTR 523

Query 506 DIENDPINCWFKAGWRPLRIREDKDTPNVYTTFAGVFKSIEDNINFDTIAKMFPKK 561

W+ LRIREDK PN ++TF V SI I DT+ + K+

Sbjct 524 T-------------WKILRIREDKAFPNAHSTFIAVCNSILMPITKDTLYYVVEKR 566
