# Supplementary figures and images for: A Metazoan/Plant-like Capping Enzyme and Cap Modified Nucleotides in the Unicellular Eukaryote Trichomonas vaginalis
Source: PLoS Pathog. 2010 Jul 15;6(7):e1000999. doi: 10.1371/journal.ppat.1000999 (PMC2904801; doi:10.1371/journal.ppat.1000999)

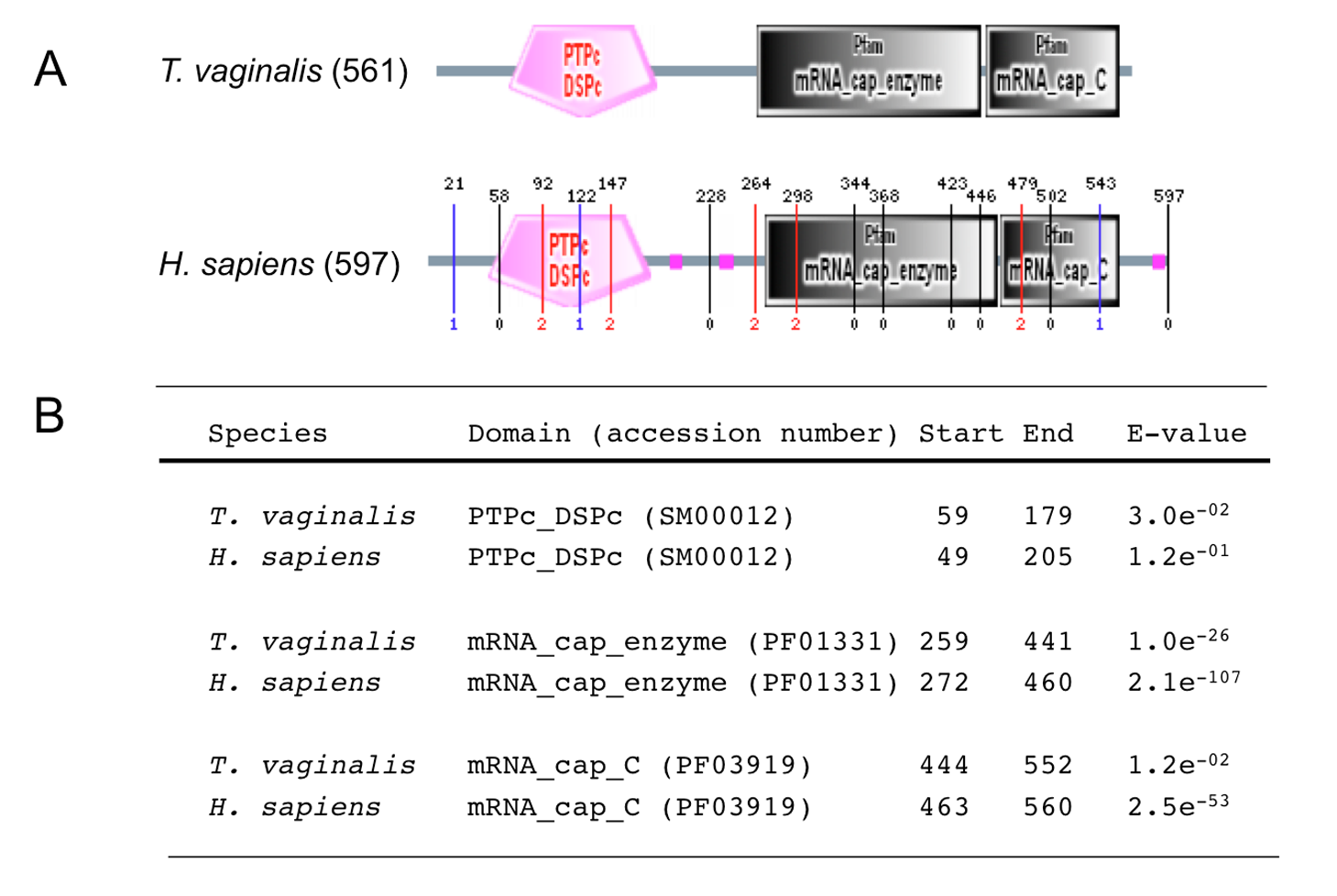

Supplement: Figure S1 — Structural organization of TvCE. A. SMART (http://smart.embl-heidelberg.de/) analyses identified the same three domains in TvCE found in other TPasePL-GTase proteins. The corresponding structural organization for the human sequence is shown for comparison. In the human sequence the pink polygon corresponds to a segment of low compositional complexity and vertical bars corresponds to intron positions. Values below bars indicate intron phase and values above the bars indicate the corresponding amino acid positions. Overlapping domains identified by SMART are not indicated. B. Position and e-values for the three identified domains (TPase: PTP_DSPc: GTase: mRNA cap enzyme; C: cap C domain) in the parasite and humans sequences with the most significant scores are tabulated for comparison. (0.35 MB TIF) [file ppat.1000999.s002.tif]

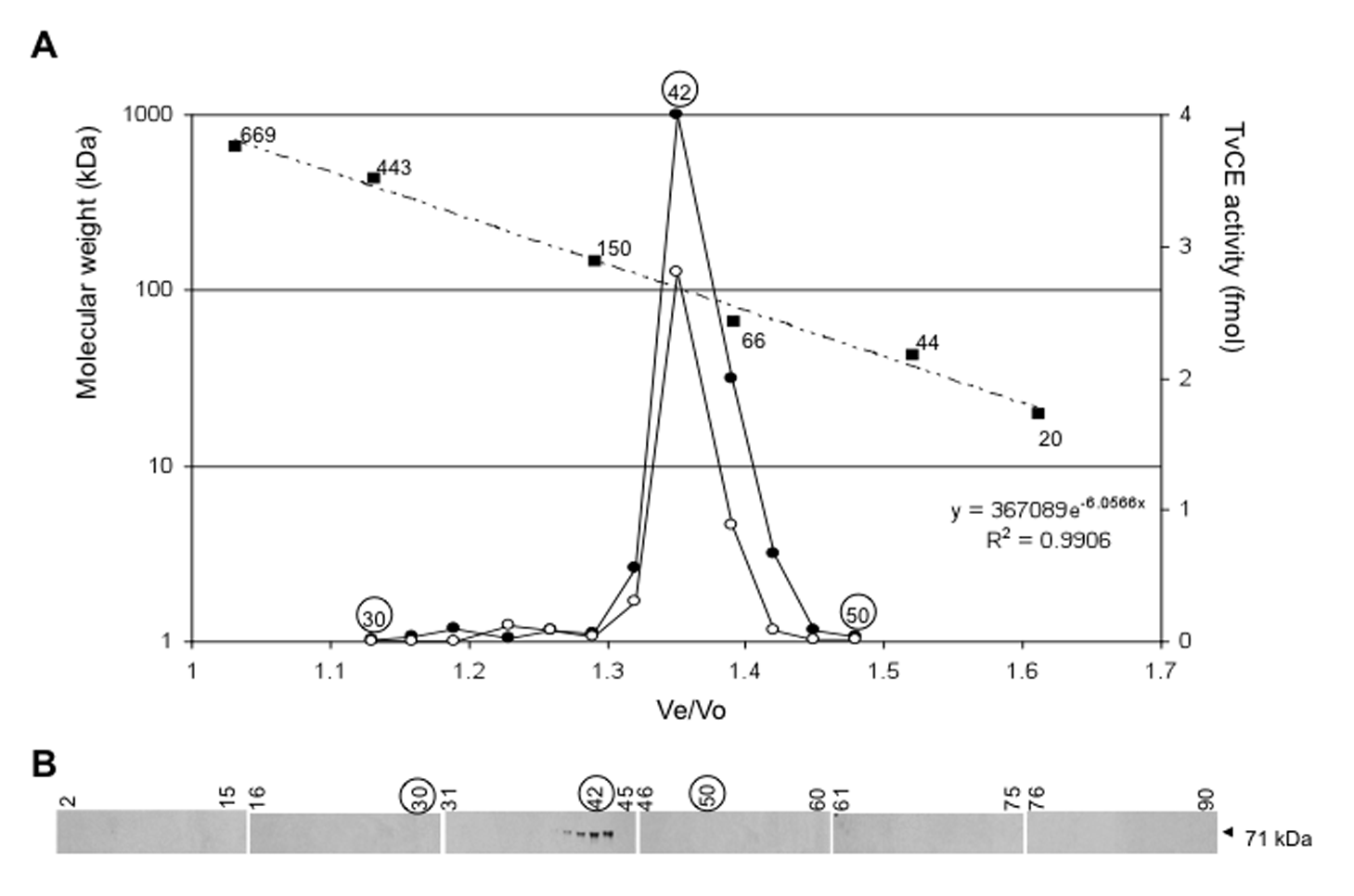

Supplement: Figure S2 — Determination of the molecular weight of the active form of TvCE. The recombinant TvCE was subjected to size exclusion chromatography in a pre-calibrated 90–10 cm Superdex™ 200 column (GE). Molecular weight (MW) markers (Sigma): Soybean trypsin inhibitor (20 kDa), chicken egg albumin (∼44 kDa), bovine serum albumin (66 kDa), alcohol dehydrogenase (∼150 kDa), apoferritin (443 kDa) and thyroglobulin (∼669 kDa). For MW curve standardization, excluded volume (Vo) was estimated by dextran blue, and elution volume (Ve) for each protein was collected and calculated. A 2 ml mix containing 0.5 mg of each protein, 50mM Tris pH 6.0; 200 mM NaCl, 5mM DTT; 10% glycerol was loaded into the column. Ninety five fractions (2 ml each) were collected at 0.2 ml/min. A. MW curve and TvCE activity. The linear regression of the MW curve (dotted line) and its equation are shown along with GTase (filled circle) and TPase (open circle) activity curves. Each MW marker is indicated by size (filled square). The activity peak and the first and last fractions tested for TvCE activity are indicated by a circled number which corresponds to the fraction number (fractions 30–50). The y axis indicates the molecular weight values (left) and GTase and TPase activities (right), and the x axis indicates Ve/Vo. B. Western blot analysis of TvCE after size fractionation. Five microliters of fractions 2 to 90, as indicated, were analyzed by western blot with HisProbeTM-HRP (Pierce). The peak fraction 42, and every other fraction from 30 (∼440 kDa) to 50 (∼50 kDa), denoted by circles, were examined for TvCE activity as described in Methods. (4.26 MB TIF) [file ppat.1000999.s003.tif]

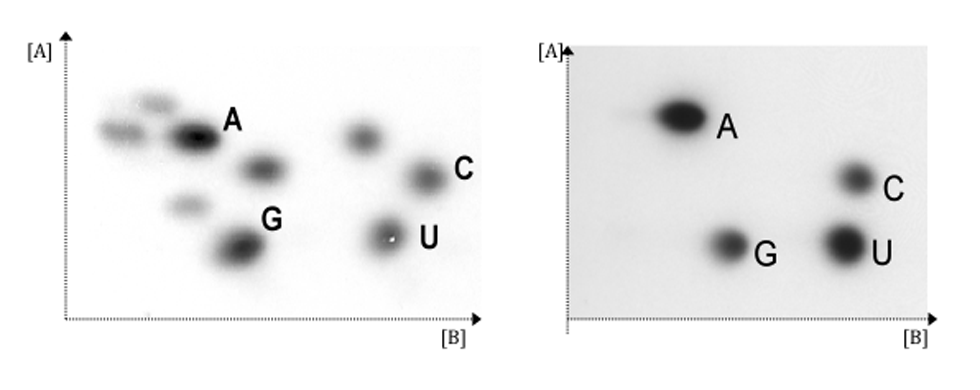

Supplement: Figure S3 — 2D-TLC resolution of P1-digested mRNA purified from T. vaginalis. Left panel, in vivo-labeled mRNA was prepared as described in Methods except that three rounds of oligo(dT) chromatography was performed but the final step of anti-TMG precipitation was omitted. Right panel, mRNA was prepared exactly as described in Methods. The final mRNA sample from each approach was then precipitated with ethanol and digested with nuclease P1 to completion. A total of 10,000 cpm of the digested RNA was resolved by 2D-TLC, as decribed in Methods, and resolved with solvents A and B (43) as indicated. Cold unmodified nucleotides (A, C, G and U) were loaded in each plate as denoted. As observed, even with one additional round of purification by oligo(dT) chromatography but without anti-TMG immunoprecipitation (left panel), the complete digestion of this mRNA sample produces the four unmodified nucleotides plus additional six detectable spots which seem to correspond to hypermodified nucleotides found in tRNAs as compared to 2D-TLC maps previously reported (43). However, when a final step of anti-TMG immunoprecipitation was included for the purification of mRNAs (right panel), the complete digestion of this sample resulted in the apperance of the four unmodified ribonucleotides only, the typical composition of mRNA molecules in a cell. Additionaly, the relative percentage of A/T (∼70%) is in accordance with A/T content of genes in this organism (29). (1.17 MB TIF) [file ppat.1000999.s004.tif]
