# Supplementary material for: A Metazoan/Plant-like Capping Enzyme and Cap Modified Nucleotides in the Unicellular Eukaryote Trichomonas vaginalis
Source: PLoS Pathog. 2010 Jul 15;6(7):e1000999. doi: 10.1371/journal.ppat.1000999 (PMC2904801; doi:10.1371/journal.ppat.1000999)
Supplement: Figure S4 — Alternative protein phylogenetic analyses of TvCE. A. Global phylogeny of GTases with the same alignment used to generate phylogeny depicted in Figure 8 with the iridovirus sequence removed. The arrow indicates the branch leading to all TPasePL-GTase configured sequences. B. Global phylogeny of GTases with the same alignmnent used to generate phylogeny depicted in Figure 8 with the Giardia sequence removed. The arrow indicates the branch leading to all TPasePL-GTase configures sequences. C. Same as in A with the Giardia sequence further removed. D. Phylogeny of TPasePL-GTase configured sequences with the same alignment used to generate the phylogeny depicted in Figure 9 with the iridovirus removed. In all trees the species names are abbreviated with the first three letters of the genus and species name (full names are listed in supplementary Table S1) and the LG model with G was used. Shown values are bootstrap proportions (%, 100 replicates), values >50% are shown. The alpha shape parameter was optimized first and fixed for the bootstrap analyses with NNI and TBR branch swapping for further optimizations. Scale bars represent the inferred number of changes per site. (0.20 MB PDF) [file ppat.1000999.s005.pdf]

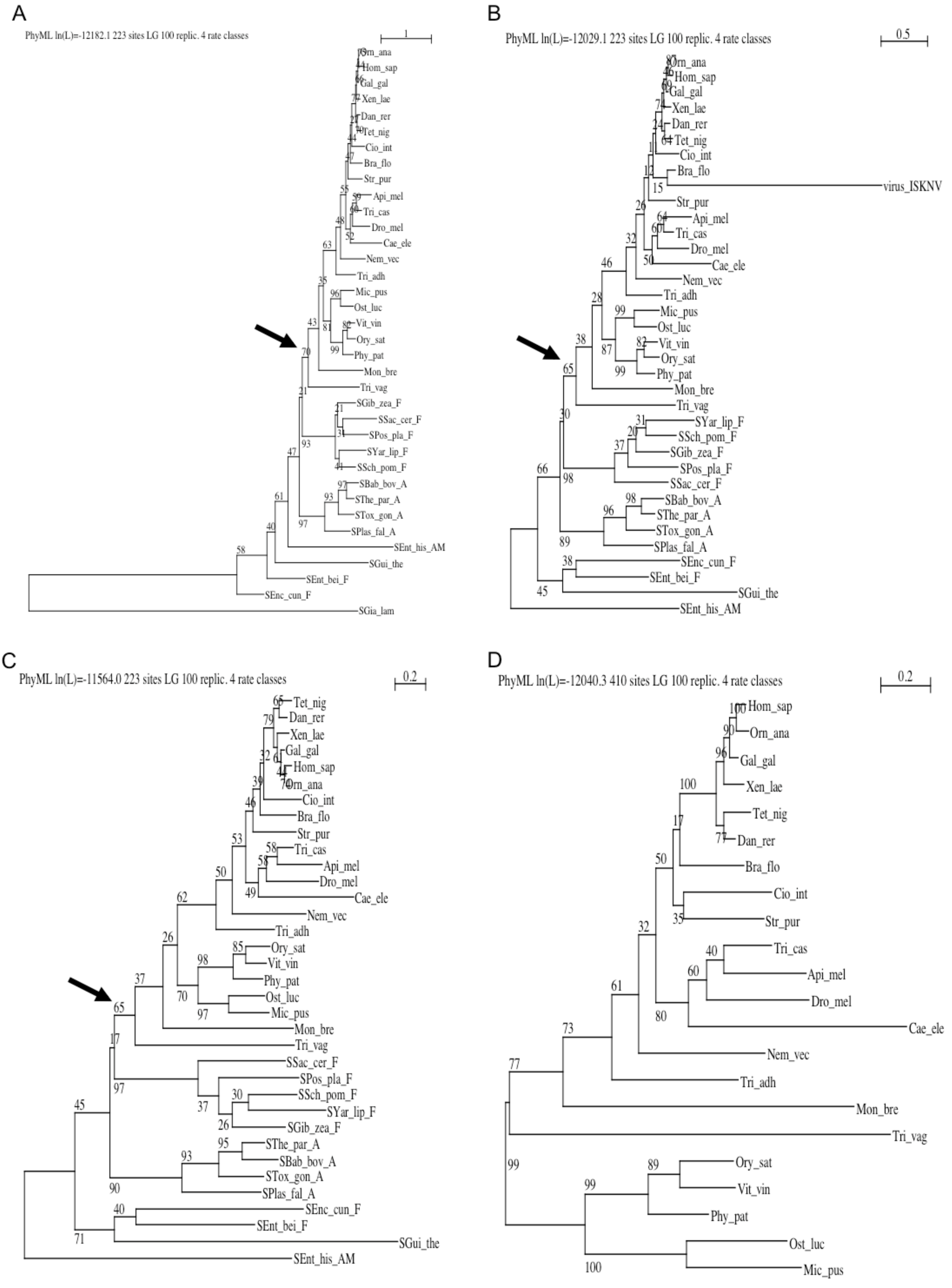
A
PhyML ln(L)=-12182.1 223 sites LG 100 replic. 4 rate classes
1
Orn_ana
Hom_sap
Gal_gal
Xen_lae
Dan_rer
Tet_nig
Cio_int
Bra_flo
Str_pur
Api_mel
Tri_cas
Dro_mel
Cae_ele
Nem_vec
Tri_adh
Mic_pus
Ost_luc
Vit_vin
Ory_sat
Phy_pat
Mon_bre
Tri_vag
SGib_zea_F
SSac_cer_F
SPos_pla_F
SYar_lip_F
SSch_pom_F
SBab_bov_A
SThe_par_A
STox_gon_A
SPlas_fal_A
SEnt_his_AM
SGui_the
SEnt_bei_F
SEnc_cun_F
SGia_lam
B
PhyML ln(L)=-12029.1 223 sites LG 100 replic. 4 rate classes
0.5
Orn_ana
Hom_sap
Gal_gal
Xen_lae
Dan_rer
Tet_nig
Cio_int
Bra_flo
virus_ISKNV
Str_pur
Api_mel
Tri_cas
Dro_mel
Cae_ele
Nem_vec
Tri_adh
Mic_pus
Ost_luc
Vit_vin
Ory_sat
Phy_pat
Mon_bre
Tri_vag
SYar_lip_F
SSch_pom_F
SGib_zea_F
SPos_pla_F
SSac_cer_F
SBab_bov_A
SThe_par_A
STox_gon_A
SPlas_fal_A
SEnc_cun_F
SEnt_bei_F
SGui_the
SEnt_his_AM
C
PhyML ln(L)=-11564.0 223 sites LG 100 replic. 4 rate classes
0.2
Tet_nig
Dan_rer
Xen_lae
Gal_gal
Hom_sap
Orn_ana
Cio_int
Bra_flo
Str_pur
Tri_cas
Api_mel
Dro_mel
Cae_ele
Nem_vec
Tri_adh
Ory_sat
Vit_vin
Phy_pat
Ost_luc
Mic_pus
Mon_bre
Tri_vag
SSac_cer_F
SPos_pla_F
SSch_pom_F
SYar_lip_F
SGib_zea_F
SThe_par_A
SBab_bov_A
STox_gon_A
SPlas_fal_A
SEnc_cun_F
SEnt_bei_F
SGui_the
SEnt_his_AM
D
PhyML ln(L)=-12040.3 410 sites LG 100 replic. 4 rate classes
0.2
Hom_sap
Orn_ana
Gal_gal
Xen_lae
Tet_nig
Dan_rer
Bra_flo
Cio_int
Str_pur
Tri_cas
Api_mel
Dro_mel
Cae_ele
Nem_vec
Tri_adh
Mon_bre
Tri_vag
Ory_sat
Vit_vin
Phy_pat
Ost_luc
Mic_pus
