# Supplementary material for: A Metazoan/Plant-like Capping Enzyme and Cap Modified Nucleotides in the Unicellular Eukaryote Trichomonas vaginalis
Source: PLoS Pathog. 2010 Jul 15;6(7):e1000999. doi: 10.1371/journal.ppat.1000999 (PMC2904801; doi:10.1371/journal.ppat.1000999)
Supplement: Table S1 — Full names of species used in phylogenetic analyses and accession numbers for all gene sequences used in the work described here. (0.05 MB DOC) [file ppat.1000999.s006.doc]

**Supplementary Table 1.** Full names of species used in phylogenetic analyses and accession numbers for all gene sequences used in the work described here.

| Species Accession number |
| --- |
| **1. TPasePL-GTase** |
| *Apis mellifera*^1^ XP_397436.3 |
| *Branchiostoma floridae*^1^ XP_002225005.1 |
| *Caenorhabditis elegans*^1, 2^ NP_001020980.1 |
| *Ciona intestinalis*^1^ XP_002130836.1 |
| *Danio rerio*^1^ NP_998032.1 |
| *Drosophila melanogaster*^1,2^ NP_572952.1 |
| *Gallus gallus*^1^ XP_419843.2 |
| *Homo sapiens*^1,2^ NP_003791.3 |
| *Micromonas pusilla*^1^ EEH55839.1 |
| *Monosiga brevicollis*^1^ XP_001749658.1 |
| *Mus musculus*^2^  NP_036014.1 |
| *Nematostella vectensis*^1^ XP_001632157.1 |
| *Ornithorhynchus anatinus*^1^ XP_001513473.1 |
| *Oryza sativa*^1,2^ NP_001066345.1 |
| *Ostreococcus lucimarinus*^1^ XP_001416488.1 |
| *Physcomitrella patens*^1^ XP_001754697.1 |
| *Strongylocentrotus purpuratus*^1^ XP_782740.1 |
| *Tetraodon nigroviridis*^1^ CAG09212.1 |
| *Trichomonas vaginalis*^1,2^ XP_001327945.1  TrichDB accession^1,2^ TVAG_187730 |
| *Trichoplax adhaerens*^1^ XP_002114529.1 |
| *Tribolium castaneum*^1^ XP_972171.1 |
| Virus - Infectious spleen  and kidney necrosis virus^1^ NP_612286.1 |
| *Vitis vinifera*^1^ XP_002277969.1 |
| *Xenopus laevis*^1^ NP_001084232.1 |
| **2. GTase (functional with TPaseMDP)** |
| *Babesia bovis*^1^ XP_001611740.1 |
| *Crithidia fasciculata*^2^ AAC27927  *Cyanidioschyzon merolae*^1^ CMA035C (KEGG database)  *Dictyostelium discoideum*^1^ XP_636333 |
| *Encephalitozoon cuniculi*^1,2^ XP_955593.1 |
| *Entamoeba histolytica* ^1^ XP_652201.2 |
| *Enterocytozoon bieneusi*^1^ XP_001827843.1 |
| *Giardia lamblia*^1,2^ XP_001710153.1 |
| *Gibberella zeae*^1^ XP_387127.1 |
| *Guillardia theta*^1^ XP_001713326.1 |
| *Plasmodium falciparum*^1,2^ XP_001348317.1 |
| *Postia placenta*^1^ XP_002472334.1 |
| *Saccharomyces cerevisiae*^1,2^ NP_011385.1 |
| *Schizosaccharomyces pombe*^1^ NP_595708.1 |
| *Theileria parva*^1^ XP_764918.1 |
| *Toxoplasma gondii*^1^ XP_002370325.1 |
| *Trypanosoma brucei*^2^ XP_829514.1 |
| *Yarrowia lipolytica*^1^ XP_503479.1 |

1 – Genes used in the phylogenetic analyses shown in Fig. 8 or Fig. 9.

2 - Genes used in the alignments shown in Fig 1.
